# Breathing new life into tissue engineering: exploring cutting-edge vascularization strategies for skin substitutes

**DOI:** 10.1007/s10456-024-09928-6

**Published:** 2024-06-06

**Authors:** M. Zohaib Iqbal, Mahrukh Riaz, Thomas Biedermann, Agnes S. Klar

**Affiliations:** 1 Tissue Biology Research Unit, Department of Surgery, University Children’s Hospital Zurich, Wagistrasse 12, CH-8952 Zurich, Switzerland; 2 Children’s Research Center, University Children’s Hospital Zurich, Zurich, Switzerland; 3 University of Zurich, Zurich, Switzerland

**Keywords:** Angiogenesis, Blood vessels, Dermal substitutes, Endothelial cells, Mesenchymal stem cells, Scaffolds, Skin defect, 3D bioprinting

## Abstract

Tissue-engineered skin substitutes (TESS) emerged as a new therapeutic option to improve skin transplantation. However, establishing an adequate and rapid vascularization in TESS is a critical factor for their clinical application and successful engraftment in patients. Therefore, several methods have been applied to improve the vascularization of skin substitutes including (i) modifying the structural and physicochemical properties of dermal scaffolds; (ii) activating biological scaffolds with growth factor-releasing systems or gene vectors; and (iii) developing prevascularized skin substitutes by loading scaffolds with capillary-forming cells. This review provides a detailed overview of the most recent and important developments in the vascularization strategies for skin substitutes. On the one hand, we present cell-based approaches using stem cells, microvascular fragments, adipose tissue derived stromal vascular fraction, endothelial cells derived from blood and skin as well as other pro-angiogenic stimulation methods. On the other hand, we discuss how distinct 3D bioprinting techniques and microfluidics, miRNA manipulation, cell sheet engineering and photosynthetic scaffolds like GelMA, can enhance skin vascularization for clinical applications. Finally, we summarize and discuss the challenges and prospects of the currently available vascularization techniques that may serve as a steppingstone to a mainstream application of skin tissue engineering.

**Graphical abstract:**

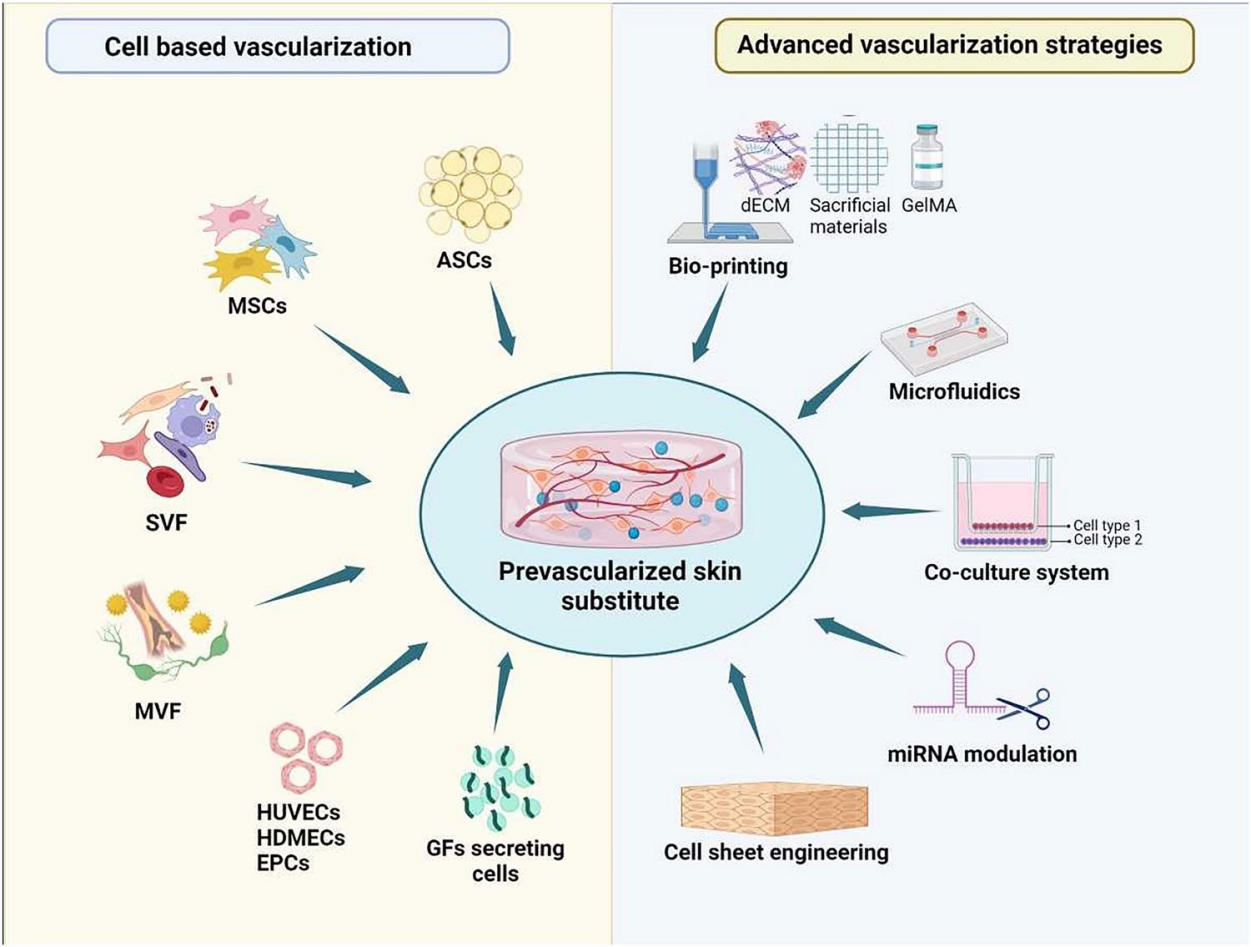

## Introduction

Full-thickness skin defects are caused by trauma, severe burn, necrotizing soft-tissue infection, and tumor resection [1]. Skin defects are usually treated by the application of split-thickness autologous skin grafts, which remain the gold standard in the clinic. However, the donor site morbidity, hyperpigmentation, itching, infection, and scarring are the major complications of this procedure [2]. Therefore, alternative therapeutic strategies are urgently needed.

The perpetual challenge of treating severe wounds has driven the exploration of skin tissue engineering, where the selection of suitable skin substitutes demands meticulous consideration of material properties. Recent technological advancements offer a wide array of materials and innovative techniques, thereby expanding the applications of skin substitutes across diverse clinical realms.

In this review, skin substitutes are defined as materials utilized to replace, mimic, or augment any skin function on damaged skin. This encompassing definition includes a spectrum of wound dressings applicable at the wound site to restore skin functionality temporarily or permanently. Examples range from skin substitutes explicitly designed for wound management to bioprinted prevascularized skin substitutes. In addition to fundamental protective functions such as water retention, antibacterial properties, and adhesion, skin substitutes are engineered to embody advanced attributes including mechanical resilience, scar resistance, and natural skin resemblance. Incorporated cells within these substitutes play a pivotal role by secreting proteins, cytokines, and growth factors (GFs) crucial for wound healing. Various cell types are selected to mimic the epidermis, dermis, and dermo-epidermal composite, thus mirroring the architecture of healthy skin. Notably, the development of increasingly multifunctional skin substitutes is gaining traction in current research endeavours. Skin tissue engineering, first introduced in the 1980s, offers alternative treatment option in plastic and reconstructive surgery. The introduction of tissue-engineered skin replacement therapies revolutionized the treatment of acute and chronic skin wounds. Previously reported experimental and clinical research imply that skin tissue engineering remarkable improved wound healing treatment and thus, may potentially represent the preferred advanced treatment option for severe skin defects in the near future [3].

Recently we [4–10] and other groups [11] have developed dermo-epidermal skin substitutes (DESS) that contain both the dermal and epidermal layers. Although DESS representatives an advanced skin replacement therapy, it still lack a vascular component. Indeed, rapid and adequate vascularization is crucial for wound oxygenation and the proper healing of skin injuries. Due to the diffusion limit, the cells farthest from the wound surface may experience hypoxia when multiple layers are combined to form a skin construct. Thus, insufficient vascularization represents a serious risk for the therapeutic application of transplanted skin substitutes, which may become infected, partially necrotic, or even demonstrate a complete graft failure [12]. Therefore, effective vascularization is a crucial requirement for the successful applications of these grafts in clinical settings.

Therefore, several methods have been employed to improve the vascularization of skin substitutes. These approaches can be divided into pro-angiogenic and prevascularization techniques [13]. Pro-angiogenic approaches aim to enhance the ingrowth of blood vessels into the implanted tissue constructs mostly by sprouting angiogenesis. However, large implants cannot be rapidly vascularized using a pro-angiogenic approach due to the slow growth rate of microvessels, which is approximately only 5 μm/h [13]. In this case, cell-based as well scaffold based (3D printing), or scaffold free (cell sheet) skin substitutes might show delayed healing. Therefore, *in vitro* prevascularization offers an alternative strategy that triggers repaid vascularization by inoculating the *in vitro* prefabricated vascular networks with blood vessels at the site of graft transplantation [14]. This strategy enhances re-vascularization and allows a prompt blood supply in a noticeably shorter time than the pro-angiogenic approach.

In the following chapters, we discuss the advantages and disadvantages of the following approaches to enhance vascularization in tissue engineering applications: (a) cell-based *in vitro* prevascularization, (b) scaffold based (3D printing), (c) scaffold-free (cell sheet) prevascularization approaches, and (d) pro-angiogenic GF-based techniques.

## Cell-based vascularization strategies for tissue-engineered skin grafts

One of the most important vascularization techniques in tissue engineering is the so called “cell-based *in vitro* prevascularization approach. This technique involves growing endothelial cells (ECs) directly within biomaterials, mostly in the presence of other cell types that support vascular network formation. [14, 15]. Following implantation, the pre-formed vascular network connects with the host vasculature, a process known as inosculation [14]. Importantly, specific topographical and biochemical properties of scaffolds can directly support the formation of a vascular network. Even in more complex tissue-engineered tissues, the implantation of pre-formed vasculature enables a rapid connection to the host vascular network through newly developed anastomoses, ensuring prompt perfusion of the implant and improved graft take chances. In contrast to non-vascularized grafts, the transplantation of prevascularized collagen I-based skin substitutes in mice stimulated the formation of functional perfused vessels, supporting the idea that rapid inoculation improves the survival of the TESS by minimizing the probability of ischemia [16].

However, the applied ECs need to meet certain criteria to be used for prevascularization in a clinical setting. They should be easy to harvest, posing little discomfort and low risk to the patient, and showing high proliferation rates *in vitro* since high numbers of cells are needed to vascularize large skin substitutes. Moreover, the cells should exhibit low immunogenic potential without any risk of developing cancer [17].

So far several EC types harvested from macro- or microvasculature have been employed for *in vitro* prevascularization approach. For example, human umbilical vein ECs (HUVECs) are generally simple to extract and maintain in culture, and thus, they represent one the most frequently applied mature EC type [17]. Due to their low immunogenicity, HUVECs are frequently employed to generate blood arteries and are excellent cell models to study EC biology.

Moreover, other primary ECs can be also extracted and cultured from various arteries and blood vessels of diverse parts of the body, and as a result, their phenotype and function may vary. For instance, artery-derived ECs are long and narrow, while vein-derived ECs are short and wide. The heterogeneity of ECs could result in variations in the functionality of the established vascular network in prevascularized grafts, such as the generation and release of vasoactive substances, production of GFs, response to endothelial mitogens, and their involvement in vessel sprouting and maturation. According to previous research, the findings from trials involving macrovascular ECs, such as HUVECs, demonstrated that such EC should not be applied in studies where microvascular bed is in focus. Along with the specific heterogeneity of EC populations, it is also important to consider the donor-to-donor differences.

 Although ECs are the main cell type involved in angiogenesis, studies have shown that EC alone *in vitro* are not sufficient to mimic physiologicalangiogenesis in tissue engineering applications. This is due to the fact that vascular structures formed by ECs alone are incomplete and prone to cell death over time. This occurs because in monocultures, ECs lose their capacity to self-assemble into tube-like structures and fail to form a complex functional vasculature [18]. Therefore, co-culture system is a common strategy to trigger vascularization by combining ECs with supporting cells. Fibroblasts, human mesenchymal stem cells (MSCs) or smooth muscle cells (SMCs) are the most prevalent types of supporting cells also known as pericytes or mural cells that can be co-culture with ECs to trigger the specific pro-angiogenic factors secretion required for survival, migration, and proliferation of ECs. Those specific cell–cell interactions are pivotal for the formation of *in vitro* capillary-like networks. According to several studies [19, 20], fibroblasts improve the mechanical properties of the extracellular microenvironment in co-culture systems by depositing matrix. This creates a scaffold for other cells to migrate and proliferate. When embedded in a Matrigel plug and implanted into mice, fibroblasts have been demonstrated to regulate the angiogenic process [19, 20]. Further, fibroblasts stimulated ingrowth of ECs from the mice and aided in the rapid implant’s vascularization.

Both two-dimensional (2D) or three-dimensional (3D) culture models can be used to generate co-culture systems in the prevascularization of tissue constructs. For a very long time, 2D systems were the standard for cell growth and studying cell–cell interactions in mono- and co-cultures. However, *in vitro* angiogenesis in 2D cell culture models do not accurately replicate the tissue architecture and physiological parameters. Importantly, heterotypic cell–cell and cell–matrix interactions in 3D hydrogel systems appear to reflect the physiological environment *in vivo* more accurately, influencing proliferation, differentiation, and signaling pathways [21].

Further, selection of an appropriate biomaterial or scaffold is also crucial because ECM supports the organization of ECs into microvessels. Moreover, GFs bind to ECM, thus influencing vascular network formation. Since various biomaterials containing distinct ECM products demonstrate different mechanical properties, important biological features like cell adhesion and migration might be affected. Additionally, other factors such as porosity architecture of a scaffold, as well as other properties including biodegradability, oxygen permeability, biocompatibility, mechanical strength, and water vapor permeability influence cell differentiation and function. In particular, the specific porosity influences the creation of a vascular network both *in vitro *and *in vivo*. The scaffold’s porous framework consists of the pore size, shape, porosity, and surface topography that permit cell migration, proliferation, cell–cell, and cell–matrix interactions.

A variety of biomaterials, including synthetic, natural, and hybrid polymers, have been explored for the fabrication of prevascularized tissue constructs. Many biodegradable, biocompatible, and non-toxic synthetic polymers have been evaluated, including polyglycolic acid (PGA), polylactic acid (PLA), polylactic-co-glycolic acid (PLGA), poly-L-lactic acid (PLLA), poly–caprolactone (PCL), polyethylene glycol (PEG), poly (vinyl alcohol) (PVA), and polyhydroxyalkanoates [18].

In summary, *in vitro* prevascularization research include the following key steps: selecting an ECs source, employing supporting cells, testing distinct scaffold materials, and adequate culture conditions. We have previously shown that seeding various capillary-forming ECs with stromal/mesenchymal cells within a hydrogel scaffold generates prevascularized dermo-epidermal skin substitutes (vascDESS) [4–7]. The *in vitro* pre-seeded cells developed into a mature capillary network, that, when transplanted into an animal, could be rapidly perfused with blood. Thus, vascDESS have the potential to be exploited for skin grafting due to the presence of a nearly physiological vascular network and, importantly, constitute a reliable *in vitro *model for dermatological research. Different ECs co-cultures can be applied for prevascularization approach. The following EC types described below have been tested and described for skin prevascularization strategies.

### Human umbilical vein ECs (HUVECs)

**Fig. 1:**
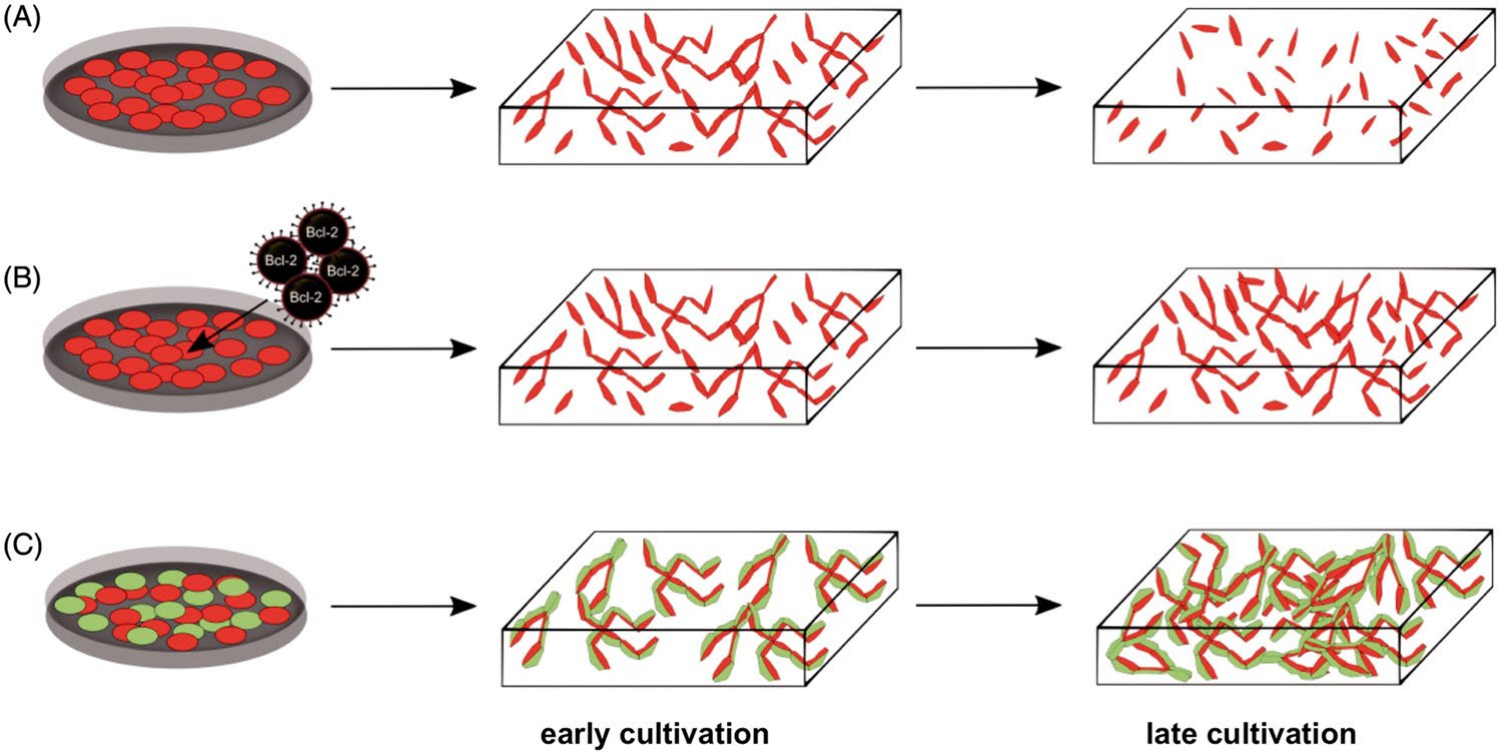
Prevascularization of skin substitutes using HUVECs. **A** To develop a capillary-like network, ECs are seeded and cultured in a 3D scaffold under optimal culture conditions. **B** ECs that have been transfected with a caspase-resistant version of Bcl-2 exhibit increased anti-apoptotic activity, survival, and tube formation. **C** For the formation of long-lasting, completely functional microvessels within the scaffold, the co-cultivation of ECs with mural cells are required [modified after 23]

HUVECs are ECs extracted from the human umbilical cord veins. The early 1970s witnessed the first successful isolation of HUVECs from human umbilical veins, which was of utmost significance in establishing human EC cultures. Indeed, HUVECs quickly established themselves as a crucial tool in vascular biology research, dominating the area to this day. Using a tissue-engineered skin substitute (TESS) model, Black et al*.* employed HUVECs to create a network resembling human capillaries in 1998 [22]. This was the pioneering work describing *in vitro* bioengineering of human microvessels using HUVECs. Further, the cells were also applied by Schechner et al*.* in a proof-of-concept study that confirmed the survival of an  engrafted bioengineered human vascular network *in vivo* in 2000 [23]. However, at the same time, HUVECs demonstrated a relatively high rate of apoptotic cell death as cultivated in 3D cultures for the development of vasculature in skin substitutes (Fig. 1A) [23]. To improve the survival of HUVECs, Schechner et al*.* [23] transfected HUVECs with caspase-resistant Bcl-2, (Fig. 1B) which increased the viability of cultivated cells and ultimately allowed them to evolve into blood-perfused microvascular networks upon transplantation into immunodeficient mice [23]. Co-cultivation of HUVECs with stromal cells is another method for preventing ECs apoptosis (Fig. 1C).

Further, in 2004, Koike et al*.* co-cultivated non-modified HUVECs and mural progenitor cells in fibronectin-type 1 collagen gels for the development of long-lasting, functional microvessels *in vivo* [24]. On the other hand, when microvessels derived from HUVECs were cultured as monocultures, they rapidly declined [24]. These findings suggest that the co-cultivation approach with mural cells is essential for the development of stable and functional microvascular networks. According to a recent publication by Kim et al*.* [25], also co-transplantation of endothelial and SMCs significantly enhances the re-vascularization and healing of skin defects.

Furthermore, before subcutaneous implantation in mice, prevascularization of fibrin constructs with HUVECs and fibroblasts accelerated the formation of anastomoses between the host and the preformed vascular network resulting in a rapid perfusion of the grafts [26]. According to some research study of Rouwkema et al*.* the development vascular structures was even possible by co-coculturing of HUVECs with osteoprogenitor cells in bone constructs before being implanted into mice [27]. The authors confirmed that* in vitro *prevascularization is a promising strategy to improve implant vascularization in bone tissue engineering. Further, Heller et al*.* [28] demonstrated the feasibility of applying HUVECs in artificially generated buccal mucosa equivalents for the reconstruction of urethral defects. Prevascularized buccal mucosa equivalents were generated in a tri-culture of primary buccal epithelial cells, fibroblasts, and microvascular ECs, using a native collagen membrane as a scaffold. The preformed of capillary-like structures became functional blood vessels through anastomosis with the host vasculature after implantation in nude mice.

These pioneer investigations on the bioengineering of vascular networks utilizing HUVECs were crucial proof-of-concept experiments that showed that preassembled human microvessels introduced into mice could connect with host vessels. Numerous subsequent investigations in the field of tissue engineering research have adopted the strategy of designing pre-assembled vascular structures before implantation in a 3D hydrogel, including attempts to vascularize engineered muscle, bone, and cardiac tissues. Future directions are not predicted to be affected by a decrease in the usage of HUVECs in vascular network bioengineering, according to trends identified in recent studies. HUVECs will probably continue to be the key ECs source in this field. However, several new ongoing developments might eventually offer new approaches and thus, reduce the application of HUVECs in tissue-engineering field. Due to the heterogeneity of ECs the growing data suggests that the endothelium governs many regenerative processes in an organ/tissue specific-way [29]. Therefore, it is recommended to use organ/tissue-specific ECs, for example by differentiation of ECs from stem cells within a specific organ/tissue [29].

### Human dermal microvascular ECs (HDMECs)

Human dermal microvascular ECs (HDMECs) represent another extensively studied source of primary human ECs. HDMECs can be isolated from skin specimens, such as juvenile foreskin. HDMECs have been shown to be efficient for the prevascularization of skin grafts since there are usually isolated from young/ juvenile patients. Importantly, HDMECs are essential for a wide range of events occurring in the skin such as tissue differentiation during skin development, immune cell adhesion, inflammatory responses, and wound healing.

Most investigations on bioengineering of vascular network with HDMECs have been carried out *in vitro*, while first *in vivo* tests were performed using immunodeficient mouse models. For example, Nör et al. utilized HDMECs encapsulated in Matrigel and implanted them into SCID mice using PLLA sponges in 2001. According to this study, HDMECs developed functional anastomoses with the mouse vasculature and organized them into microvessels that were visible seven to ten days after implantation and contained mouse blood cells in their lumina [30]. The study has also shown that at 21 days after implantation, mouse cells expressing perivascular smooth muscle actin covered human arteries, indicating the stability of the vasculature. Moreover, in 2002, Peters et al*.* employed HDMECs in VEGF-containing PLGA matrices. Within three days, the HDMECs-lined vessels formed immature structures, and after 14 days, they generated a complex, mature network [31]. Furthermore, interactions between HDMECs and fibroblasts are also crucial for the development of 3D vascular structures in human skin substitutes by mimicking closely the physiology of human skin. Importantly, Montano et al*.*, has reported that HDMECs can spontaneously organize within 3D fibrin-based scaffolds into organotypic vascular networks, which are stabilized by mural cells of the host tissue after transplantation [8].

**Fig. 2:**
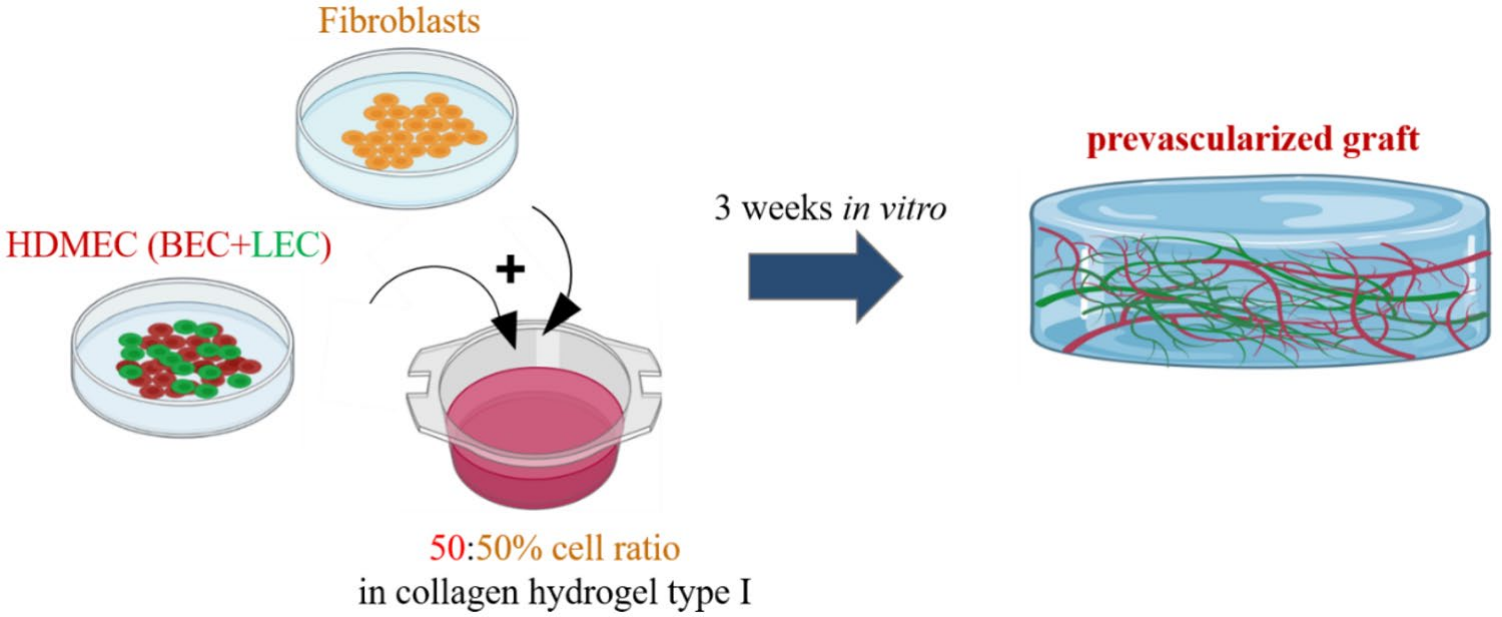
A scheme showing the preparation of prevascularized dermal hydrogel. HDMECs containing both BEC (red) and LEC (green) were combined with dermal fibroblasts in a 3D collagen type I based scaffold promotes capillary formation after 3 weeks *in vitro* culture (prepared with BioRender)

Additionally, we demonstrated the *in vitro* and *in vivo* development of human blood and lymphatic capillaries derived from the blood ECs (BEC) and lymphatic ECs (LEC) of dermal HDMECs co-cultured with dermal fibroblasts (Fig. 2) [7].

It has been shown that mural cells (pericytes) can be already attracted to the vascular structures *in vitro *as well as after transplantation [9]. The growing capillary network generates the prevascularized dermal matrix, which can be afterward used for seeding epidermal keratinocytes to create a vascDESS, which was tested in immunodeficient rats [9].

In another study, Supp et al*.* claimed that HDMECs assemble into multicellular structures and can be maintained in dermo-epidermal skin grafts *in vitro* [32]. In this study, HDMECs aggregation in the upper dermis was detected* in vitro *in proximity to the tiny pores in the dermal matrix using CD31 staining. Histology analysis of skin substitutes revealed the presence of ring-like clusters of cells matching the vascular analogs close to the dermal-epidermal junction in composite tissue constructs prepared with HDMECs. The same group also reported that HDMECs were able to organize into vascular structures in athymic mice when co-cultured with dermal fibroblasts and epidermal keratinocytes in skin substitute consisting of acellular collagen-glycosaminoglycan (GAG) substrates. However, Supp and colleagues did not demonstrate in this study any functional connection of the prevascularized substitutes to the host vasculature [32].

Further, Chrobak et al*.* used HDMECs and HUVECs to generate functional microvascular structures, co-cultured with perivascular cells, and transformed human neutrophil-like HL-60 cell line in collagen gel *in vitro* [33]. They observed continuous perfusion of human EC-containing structures, which closely mimicked the size and 3D cellular architecture of human skin microvessels. Moreover, the group applied the established prevascularized model for inflammatory response studies *in vivo*. The authors introduced a method to form open tubes of microvascular cells in a collagen gel. The observed so-called “giant” capillaries were also detected in tumors or sites of chronic inflammation and exhibit a strong barrier function, react to inflammatory stimuli, and support the adhesion of leukocytes under shear stress conditions [33].

However, there are some limitations to using HDMECs for prevascularization strategies. Those cells are mostly isolated from small skin biopsies of newborns’ foreskins. Although HDMECs isolation techniques have been improved, their use in clinical settings is still hampered by their low cell yield from small skin biopsies, a need for *in vitro* expansion, and fibroblast contamination [12]. Therefore, preparing adequate numbers of HDMECs for therapeutic tissue engineering applications is a slow process and can take up to six weeks [12]. Of note, HDMECs are also commercially accessible cells and can be obtained from allogeneic sources for *in vitro *wound assays, *in vivo* blood vessel formation, and for preclinical testing. Additionally, the improvement of serum-free culture techniques has made it possible to use these cells in a clinical setting.

## Endothelial progenitor cells (EPCs)

For numerous years, human ECs were obtained from healthy mature vasculature. However, it was widely acknowledged that this method lacked a strong clinical potential because of the morbidity caused by the removal of healthy tissues and a low proliferation potential in culture. These restrictions sparked extensive interest in developing alternative autologous human EC sources, including stem and progenitor cell sources, which may be less invasive and more reproducible. In the late 1990s a subset of EPCs, that circulate in human peripheral blood and give rise to mature ECs in culture, are identified. Undoubtedly, for therapeutic applications, the discovery of blood-derived EPCs represented a chance to obtain the autologous ECs without using any invasive methods. EPCs can be extracted from adult peripheral blood non-invasively and they demonstrate increased clonogenic potential as compared to mature ECs. As a result, these cells can be rapidly multiplied in high numbers for usage in clinical settings [34].

Human EPCs exhibit all the typical ECs markers, including the expression of VE-Cadherin, CD31, and vWF, absorption of low-density lipoproteins (Ac-LDL), and highly specificlectin binding (Ulex europaeus agglutinin 1, UEA-1) [35, 36]. The capacity of human EPCs to develop functional circulatory networks has also been confirmed *in vivo.* Additionally, EPCs maintain their endothelial identity during extended *in vitro* expansion, demonstrating stable endothelial phenotype over time [35, 36].

In 2004, Wu et al*.* reported the first application of EPCs in the development of vascular networks in vitro [37]. In this study, cord blood-derived EPCs were incorporated *in vitro* into 3D polyglycolic acid-poly -L-lactic acid scaffolds containing human SMCs to generate human microvessels that were evenly distributed throughout the construct. This study confirmed the potential of EPCs for establishing microvascular networks inside tissue-engineered constructs [37]. Other study demonstrated that EPCs have great vascular network-forming ability in comparison to vessel-derived ECs (including HUVECs) when applied in collagen hydrogel *in vitro* [35]. Furthermore, human osteoblasts and human EPCs were co-cultured *in vitro* by Fuchs et al., who showed that the EPCs developed more robust and highly complex microvessel-like structures than the HUVECs did [38].

Further, Shepherd and his colleagues first proposed the use of human EPCs to repopulate decellularized tissues [39]. Specifically, cord blood-derived EPCs were seeded into decellularized human skin substitutes, and the grafts were then implanted into mice for 21 days. The study demonstrated that EPCs incorporated into the graft vasculature that connected rapidly to underlying host blood vessels [39]. This was one of the earliest *in vivo* examples of the successful application of human EPCs infor bioengineering of the human vascular network [39]. Furthermore, Melero-Martin et al. conducted studies where human EPCs and human saphenous vein SMCs were co-seeded in Matrigel and delivered subcutaneously into immunodeficient nude mice. A complex network of lumenized structures was found after one week, which were lined by human EPCs containing murine erythrocytes, revealing the successful establishment of functional anastomoses with the host vasculature [40]. It should be noted that this investigation demonstrated the viability of using both adult peripheral blood and umbilical cord blood as potential sources of EPCs, even though Au et al. reported later that only the capillaries generated by cord blood-derived EPCs proved to be substantially long-lasting [36]. Furthermore, a study published by Yoder et al. revealed that human EPCs, when incorporated into a collagen/fibronectin hydrogel construct, were capable of forming a perfused network of blood vessels upon implantation into NOD/SCID mice [41]. This groundbreaking finding added to the growing body of evidence supporting the endothelial lineage origin of EPCs. This work was significant because it provided evidence against the previously proposedhypothesis that EPCs might have a myeloid origin. Before this study, there was debate within the scientific community regarding the exact lineage and origin of EPCs.

Furthermore, it demonstrated for the first time that human EPCs can form vascular networks in vivo without the contribution of exogenous perivascular cells. Even though, tissue grafts with only EPCs are technically possible, later studies demonstrated that the microvascular density obtained through EPCs without the use of mural cells is significantly lower than that obtained with perivascular support. Together, these results show that autologous ECs derived from human peripheral blood may be the most source for future skin tissue engineering applications.

However, one significant hurdle for EPCs application is their low abundance in adult tissues, which poses a challenge for their therapeutic application. EPCs represent just a very small portion of the circulating cells in adult human peripheral blood, amounting to around 0.05 to 0.2 cells/ml, this is roughly 15-fold less than in umbilical cord blood [42]. The isolation of adult EPCs has proven to be particularly difficult because of this low frequency and the absence of a characteristic markers. Concerns about donor variability have also been raised, and several investigations have shown that a significant part of adult subjects, both healthy and unhealthy with coronary artery disease, type two diabetes, and age-related macular degeneration, lack EPCs. Unfortunately, it is not currently known how EPCs are released into circulation or how this process changes with age.

Therefore, despite advancements in our understanding of EPC biology and their potential applications in regenerative medicine, challenges remain in harnessing their therapeutic potential. Due to their scarcity, it is difficult to develop standardized protocols for EPC-based therapies that may limit their widespread clinical application. Furthermore, variability in EPC populations among individuals, as well as changes in EPC function with age and disease states, further complicate their therapeutic potential. These factors underscore the importance of continued research to elucidate the mechanisms regulating EPC biology and identify strategies to enhance their therapeutic efficacy.

Therefore, currently, ongoing efforts are continuing to optimize EPC isolation, characterization, and genetic manipulation to overcome current limitations. Advances in stem cell biology, tissue engineering, and regenerative medicine are driving progress toward unlocking the full therapeutic potential of EPCs.

### Stromal vascular fraction (SVF)

The lack of sufficient numbers of ECs isolated from autologous sources and their low clonogenic potential continue to hinder the use of mature ECs in clinical settings. Therefore, it is necessary to focus on alternative cell sources, which can enhance the vascularization by providing cells in with high proliferation potential and promoting neo-vascularisation* in vivo*. In this respect, adipose tissue emerged as a rich source of cells for regenerative medicine. In particular, the stromal vascular fraction (SVF) of white adipose tissue (WAT) represents an abundant and easily accessible source of autologous cells. SVF is a heterogeneous population that comprises preadipocytes, stromal cells, ECs, multipotent stem and progenitor cells, and pericytes [4]. Accordingly, 1.6 − 0.9 × 10^5^ of nucleated cells can be routinely isolated from 1 ml of a fat liposuction biopsy and 1 − 0.55 × 10^5^ nucleated cells from 1 g of an excision biopsy [4].

Recently, SVF has been implemented in many wound healing applications due to the presence of multipotent stem/progenitor populations. Further, SVF cells demonstrate high angiogenic potential due to the release of pro-angiogenic GFs by ECs resulting in spontaneous capillary development and remodeling in dermal substitutes both *in vitro* and *in vivo* [4, 5]. Additionally, the proportion of stem cells and SVF proliferation ability are not substantially affected by the age of the donor [10].

**Fig. 3:**
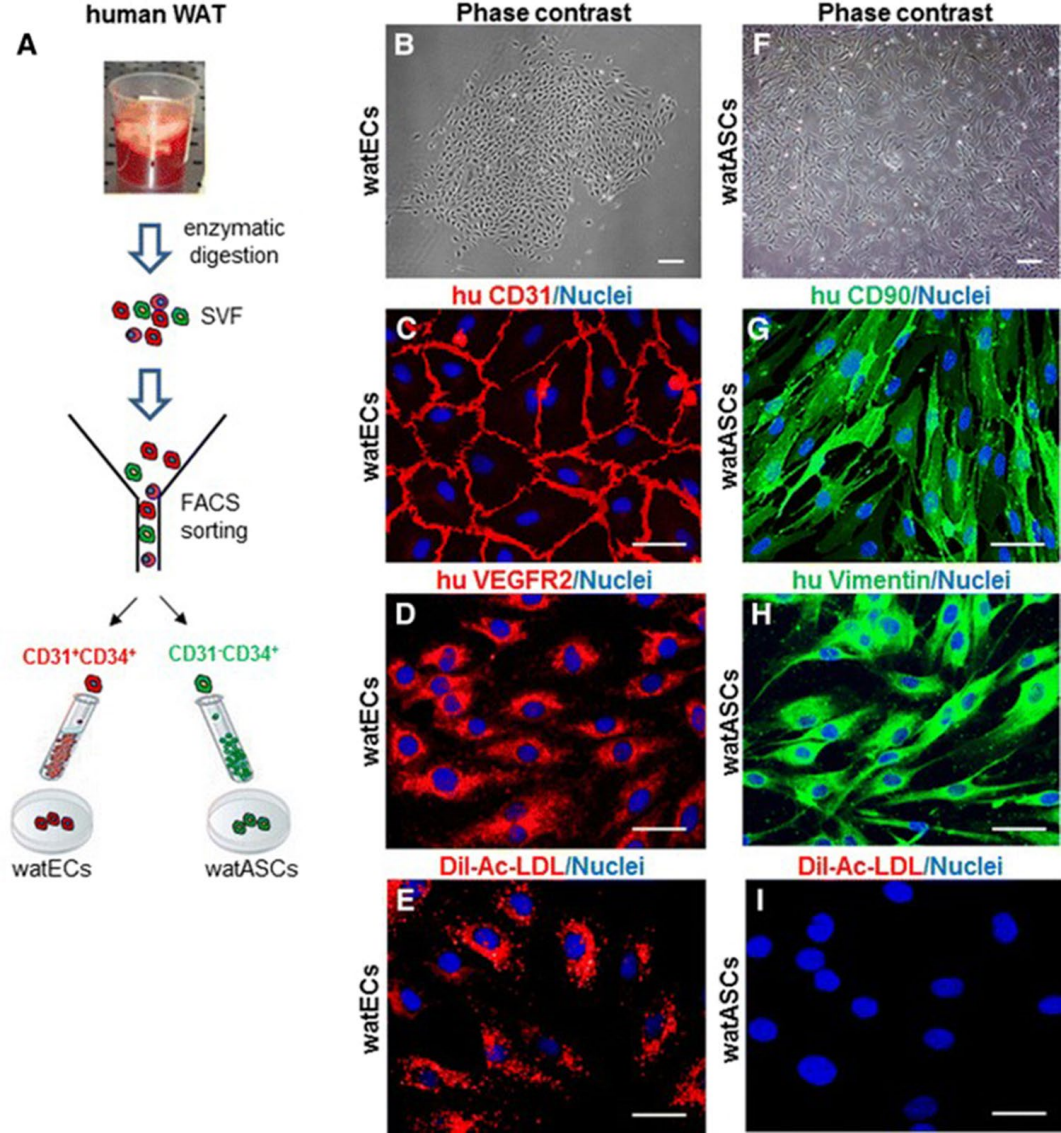
Analysis of endothelial cells (watECs) and adipose stromal cells (watASCs) of SVF. **A** Isolation and FACS sorting of watECs (red) and watASCs (green). Phase contrast microscopy showing cobblestone pattern of (**B**) watECs and spindle-shaped pattern of (**F**) watASCs. **C–E** Immunofluorescence staining of watECs showing positive expression of CD31, VEGFR2 and Dil-Ac-LDL. **G–I** Immunofluorescent evaluation of watASCs showing positive staining of CD90 and vimentin and negative for Dil-Ac-LDL. Scale bars: **B, F**: 200 µm; **C–E, G–I**: 50 µm [modified after 5]

Furthermore, we showed that, in comparison to alternative EC sources, SVF cells were the more appropriate choice for generating prevascularized skin substitutes. Whereas freshly harvested SVF contains already both—endothelial and stromal cells at a specific 1:1 ratio and in high numbers, HDMECs need to be combined with dermal fibroblasts to form capillaries [4, 5]. With this regard, the SVF contains an advantageous cell source (Fig. 3) containing both CD31^+^/CD34^+^ white adipose ECs (watECs) and CD31^+^CD34^−^ white adipose ASCs (watASCs) [4, 5].

**Fig. 4:**
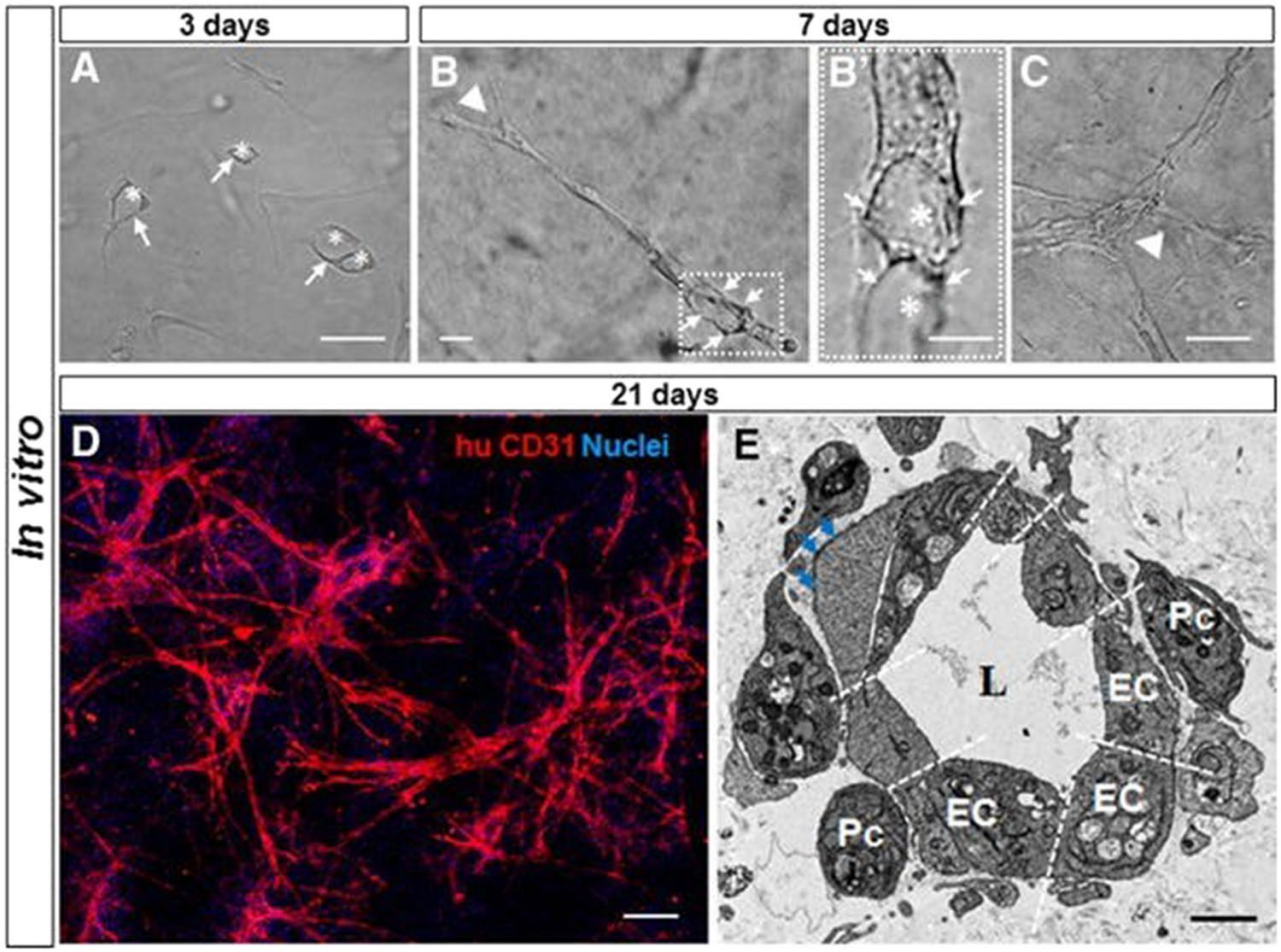
*In vitro* analysis of bioengineered capillaries. **A–C** Light microscopy of SVF-derived watECs cultured in 3D hydrogels for 3–7 days to visualize the presence of vacuolar structures (3 days) and their fusion into branched capillaries (7 days) **D** CD31 staining (red) of hydrogels after 21 days of culturing revealed a network of well-connected capillaries. **E **Electron microscopy confirmed the location of lumen (L) surrounded by several ECs and pericytes (Pc) and presence of basement membrane (blue arrows). Scale bars: **A–C** 50 µm; **D** 100 µm; **E** 1 µm [modified after 5]

Besides the cells, the selection of a specific scaffold is crucial for promoting new vessel formation and restoring and maintaining the specific tissue functions. Accordingly, distinct 3D scaffolds were designed for skin applications based on the physical and chemical properties to retain specific skin cell functions. We and others have demonstrated that the SVF can be cultured in collagen I and fibrin-based scaffolds [4, 5, 43]. Importantly, those 3D biomaterials supported the development of capillaries both *in vitro* and *in vivo*. For example, in hydrogel-based dermo-epidermal skin grafts, Klar et al*.* [5] showed that the heterogeneous SVF cell populations efficiently generated a mature microvasculature. Within 7 days, ECs developed branched and elongated capillary-like structures, and vascular lumen (white asterisks) (white arrowhead in Fig. 4). Furthermore, cells generated sprouts that dispersed until they anastomosed to form networks. The typical diameter of *in vitro* pre-formed capillaries was approximately 10 µm. After 21 days in culture, human CD31 staining revealed that a complex network of interconnected capillaries had developed (Fig. 4).

**Fig. 5:**
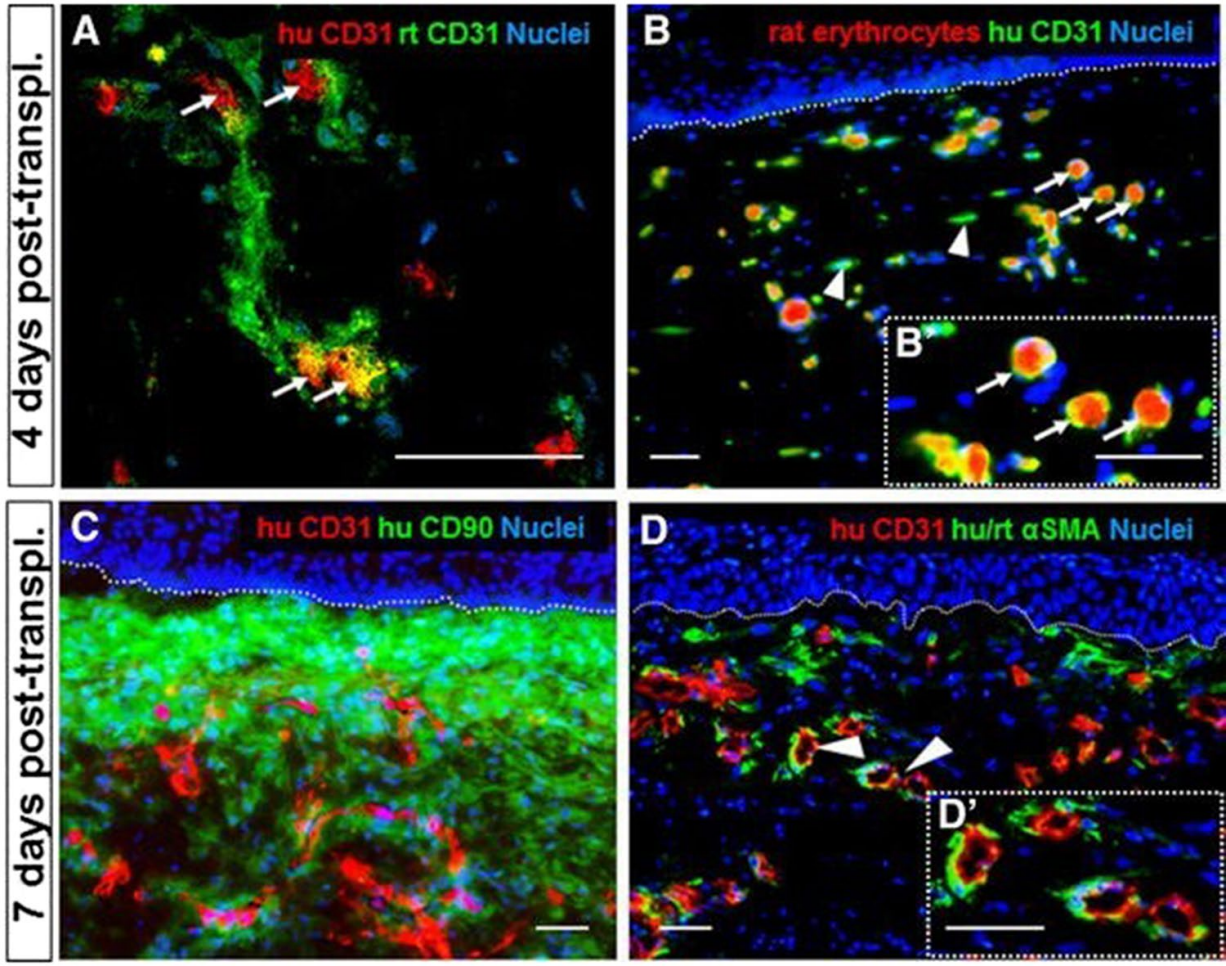
*In vivo* analysis of SVF seeded DESS **A** Immunofluorescence staining of DESS showing a connection between human capillaries (red) and rat capillaries (green) 4 days after transplantation. **B** Blood perfusion of capillaries of transplanted capillaries is confirmed by visibility of rat erythrocytes (red) inside their lumina. **C** Co-staining of CD31 and CD90 confirming the location of the capillaries in dermal compartment 7 days after DESS transplantation. **D** Presence of pericytes around the transplanted capillaries is confirmed through human/rat αSMA (pericyte marker) staining. Scale: **A** 40 µm; **B–D** 50 µm [modified after 5]

After transplantation of 3D SVF-prevascularized skin substitute into a full-thickness wound on immune-deficient rats, we confirmed effective blood perfusion and graft-host vascular anastomoses (connections) between the human and rat vascular system (Fig. 4). Importantly, rapid blood perfusion significantly improved the epidermal and dermal regeneration of skin substitutes 4,5 (Fig. 5).

### Adipose tissue-derived microvascular fragments (MVF)

**Fig. 6:**
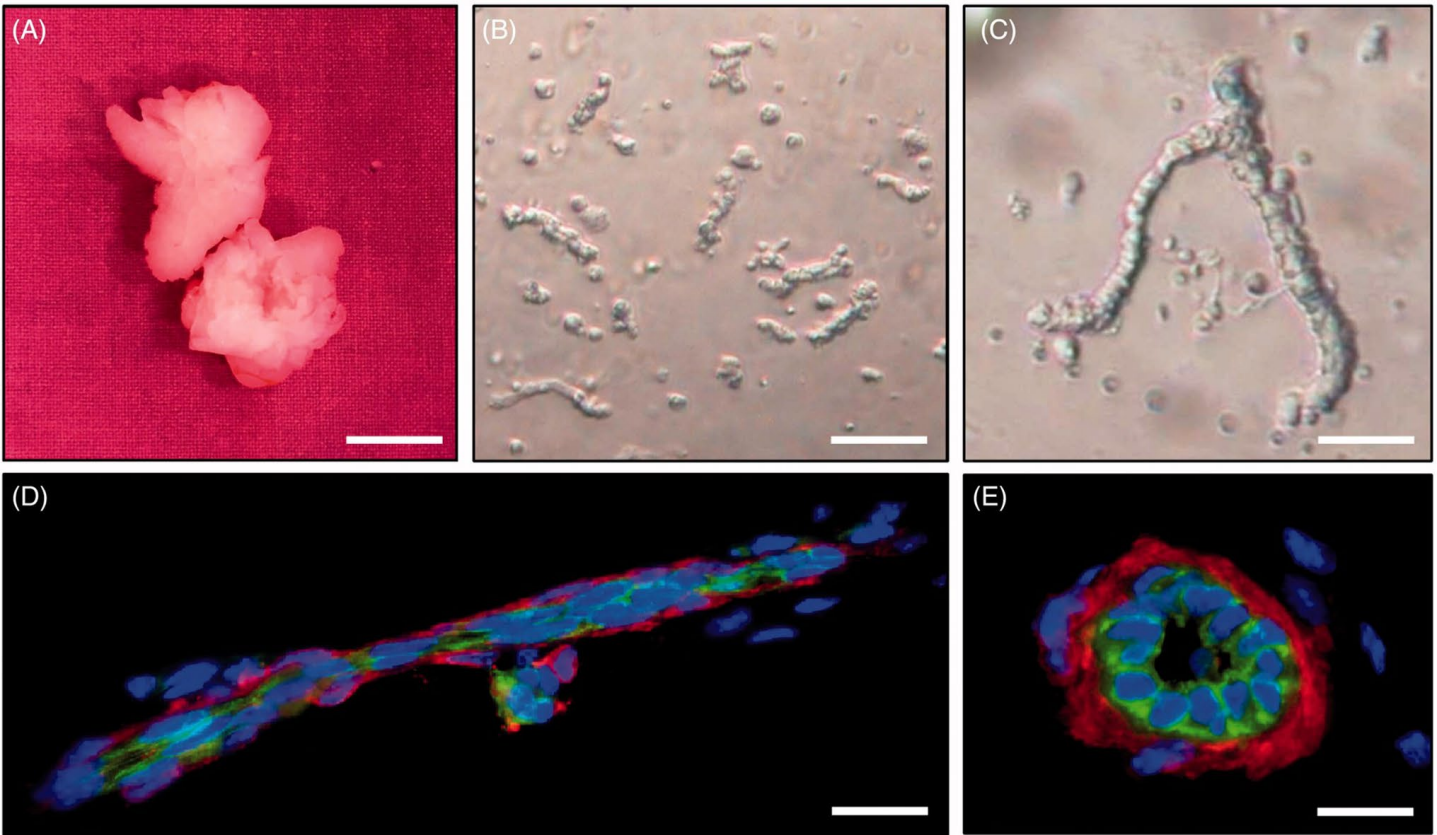
Vascularization in tissue engineering by MVF. **A** Epididymal fat pads isolated from mouse tissue. **B–C** Phase-contrast of MVF freshly isolated from fat pads. **D–E** Immunohistochemistry of MVFs showing endothelial cells (green), α-smooth muscle actin (red) and cell nuclei (blue) [modified after 47]

Microvascular fragments (MVF) refer to small segments or pieces of microvessels, which are small blood vessels. They can be isolated from various tissues, including adipose tissue, and are utilized to create vascularized tissue constructs in regenerative medicine approaches. Up to now, mostly murine SVF was used for the retrieval of adipose tissue-derived MVF due to its abundance and minimally invasive isolation procedure. Mechanical mincing and short-term enzymatic digestion can separate MVF from adipose tissue and provide significant quantities of MVF (Fig. 6). MVFs offer a significant benefit over traditional single cell extractions from fat tissue, like isolation of SVF, as they still display 3D capillary structures containing endothelial and stromal cells, that substantially enhance the angiogenic and regenerative potential of MVF. Laschke et al*.* characterized murine MVF *in vitro* and demonstrated that those microvascular networks were also functional after transplantation 44. Thus, by establishing connections with one another and the nearby blood vessels of the host tissue, they rapidly reassemble to mature blood-perfused microvascular networks. Accordingly, it has already been demonstrated that introducing MVF into tissue-engineered constructs such as bone, myocardium, and pancreatic islets can improve their vascularization [44].

Consequently, adipose-tissue derived MVF can be used in the future as a novel strategy for prevascularization of skin substitutes to accomplish prompt vascularization (Fig. 6) [45]. However, so far only murine MVFs have been characterized and applied for regenerative approaches.

Some studies investigated the specific cellular and phenotypical characteristics of MVF. According to one flow cytometric study describing murine MVF, several cell types obtained from MVF expressed stem cell surface markers, such as the marker combination Sca-1/VEGFR-2 for EPCs and CD29, CD44, CD73, CD90, and CD117 for MSCs. Thus, murine MVF contain a high proportion of multipotent MSCs, which sustain the differentiation, proliferation, and survival of MVF. Furthermore, the proliferation and capability of neurogenic, osteogenic, and adipogenic differentiation are higher in murine MVF-associated stem cells. Furthermore, Sato et al*.* reported that the myofibroblastic cells quickly condense into a monolayer in culture, which stimulates the capillary fragments on top to grow into new microvascular networks [46].

Additionally, the identification of specific EC markers within MVF has led to speculation regarding their role in postnatal vasculogenesis. MVF are found to be densely populated with pro-vascularizing cells, which possess a distinctive capacity to stimulate neovascularization within adipose-derived MVF implants. These fragments, obtained through a carefully controlled digestion process with collagenase, maintain their inherent microvessel architecture, characterized by the presence of ECs enveloped by perivascular cells, and also retain their angiogenic potential. Upon seeding these MVF into a three-dimensional collagen type I matrix and subsequently implanting them subcutaneously in vivo, a remarkable progression unfolds. These fragments undergo a transformative journey, culminating in the development of a novel, mature, and intricately organized hierarchical microvascular network. What’s more, this newly formed network seamlessly integrates with the host circulation through complete anastomosis, ensuring efficient blood flow and nutrient exchange within the surrounding tissue environment [47]. In this system, the process of neovascularization occurs spontaneously, without the need for additional factors or external agents. This inherent capability arises from the abundance of pro-angiogenic cells present in adipose tissue. Moreover, the versatility of MVF allows for the creation of organotypic microvasculatures tailored to the specific needs of different tissues or organs.

For instance, research conducted by Hoying et al. revealed that microvascular networks derived from murine MVF and cultured in collagen 3D gels undergo dynamic changes over time [48]. Initially, the networks exhibit a sprouting phenotype, characterized by extensive branching and angiogenesis. However, with continued cultivation, these networks gradually evolve towards a more pruned or reduced capillary network [48]. This phenomenon highlights the adaptive nature of MVF-derived microvasculatures, which can dynamically change during prolonged cultivation. Such flexibility is crucial for tissue engineering and regenerative medicine applications, as it allows for the development of vascular networks that closely mimic the native vasculature of specific tissues or organs.

In contrast to other cell-based *in vitro* angiogenesis assays, the MVF-culture model offers the possibility to investigate angiogenesis under physiological conditions. For example, Acosta and colleagues recently exposed murine MVF to growth medium first, followed by adipogenic induction media [43]. These culture conditions induced sprouting and the formation of new microvascular networks in the murine MVF, which showed lipid droplets, increased expression of adipogenesis-related genes, and increased lipolysis. As a result, this approach may not only improve the engineering of vascularized adipose tissue but also provide a novel model system for investigating adipose tissue expansion [43].

### MSCs

The human mesenchymal stromal cells (MSCs) were first described as adherent, bone marrow-derived cells with the capacity to form colonies [49]. Later research revealed that MSCs have multilineage differentiation potential including osteogenic, adipogenic, and chondrogenic differentiation [50]. They express the cell surface markers CD73, CD90, and CD105 but are negative for CD11b, CD14, CD34, CD45, CD79a, or HLA-DR, according to the definition given by the International Society for Cellular Therapy in 2006 [51]. MSCs have been identified in almost all types of vascularized tissues, mostly as cells that are present in perivascular regions and express pericyte markers. Bone marrow, sweat glands, submandibular glands, umbilical cord, pancreas, and adipose tissue are only a few of the tissues and organs from which MSCs can be successfully isolated. Bone marrow and adipose tissue are two main sources of MSCs used for translational research [52].

In particular, MSCs can also act as a perivascular cell source for vascular network bioengineering due to the following benefits. First, minimally invasive techniques can be used to collect autologous human MSCs from compact adipose tissue and/or bone marrow biopsies. Second, commercially available media and supplements for these cells are now available as fully-defined media, which allow maintaining and growing human MSCs in culture. Third, MSCs have the capability to regenerate mesenchymal tissues in addition to their supportive function in the development of vascular networks. Thus, MSCs have been shown to be powerful tools in regenerative medicine.

Human MSCs have been used in vascular network bioengineering since the early 2000s, albeit the word “MSCs” was not widely used. In a specially designed chorioallantoic membrane model, researchers reported that co-transplantation of human preadipocytes (i.e., adipose tissue-derived MSCs) with HDMECs facilitated the early creation of a capillary network [ 53]. Human osteoblasts (i.e., bone marrow-derived MSCs) and HUVECs were combined to create heterogeneous co-spheroids in 2004 by Wenger et al*.* The study showed that the osteoblasts promoted the sprouting of HUVECs into large capillary networks inside a 3D collagen matrix [53].

Importantly, MSCs present a balanced autocrine and paracrine GF secretion profile that supports vasculogenesis and stabilizes the newly developed capillaries. Furthermore, the possibility of MSCs being employed for prevascularization depends on their source, culture conditions, media supplements, scaffold, and co-culture ratio with ECs. Ghajar et al*.* investigated EC sprouting in 3D fibrin matrix model *in vitro* using a combination of human MSCs and HUVECs [54]. Due to the high secretion of pro-angiogenic factors by MSCs, the HUVECs formed highly complex networks when co-cultured with them. Additionally, further investigations showed that the contribution of MSCs (from various origins) required maturation into cells similar to smooth muscle. These cells served as perivascular cells since they encircled the EC-lined lumen [54].

Human MSCs’ potential to enhance vascular network bioengineering was first demonstrated *in vivo* in the late 2000s. As demonstrated by Au et al. in 2008, HUVECs and MSCs from human bone marrow were used to bioengineer vasculature *in vivo* [55]. By acting as perivascular progenitor cells, the MSCs were found to effectively stabilize developing blood vessels *in vivo*. In SCID mice, the vasculature was stabilized by pericytes and functional for more than 130 days [55]. Furthermore, Melero-Martin et al*.* showed that MSCs generated from human bone marrow support human EPCs in a similar way [40]. Both findings confirmed the role of MSCs as perivascular cells that surrounded human vessels and expressed perivascular markers including α-SMA. In contrast, they demonstrated that implanting of either ECs or MSCs alone failed to result in significant vascularization. These data proved that it is possible to bioengineer vascular networks *in vivo* employing human MSCs as a source of perivascular cells [55, 56].

Furthermore, researchers demonstrated that tissue resident MSCs obtained from different tissues exhibited similar  potential toward the development of human vascular networks *in vivo*. This proved that regardless of their initial anatomical location, all MSCs possess the pro-angiogenic potential.  Furthermore, Lin et al*.* demonstrated that ECs themselves attract MSCs via paracrine signaling, confirming the mutual cooperation between both cell types. In contrast, MSCs, which were unable to engraft as perivascular cells, lost their stemness character and transformed into fibroblast-like interstitial cells [57]. Specific trophic factors from ECs, such as PDGF-BB, were proved to be essential for maintaining the perivascular nature and stem cell capabilities of MSCs *in vivo*. This study suggested that the co-implantation of ECs and MSCs is required to obtain mature and functional vascular networks by a proper stimulation of MSCs engraftment and their differentiation into perivascular cells [57].

To conclude, MSCs show a high potential to beapplied for vascular network bioengineering. However, there are several issues that have yet to be answered. This includes questions regarding donor-to-donor variability and the differences between various tissues of origin. For instance, *in vivo* studies have frequently shown lineage-restricted features, like multilineage differentiation potential, that are directly connected to their tissue of origin. Because of this, it is unknown if all MSCs have the same multilineage differentiation ability *in vivo*. Therefore, further research is needed to determine the variations among the different human MSCs sources as well as their long-term impacts on the vasculature. Certainly, increased knowledge of the biological characteristics of MSCs will lead to a multiple applications of these cells in the field of vascularization [57].

### ASCs

Adipose tissue-derived stem cells (ASCs) present a compelling and valuable resource for regenerative skin engineering endeavours. Extensive investigations, both *in vitro* and *in vivo*, confirmed their capacity to undergo differentiation into diverse lineages of skin cells. Additionally, ASCs are acknowledged for their robust potential in skin regeneration. This is attributed to their capacity to secrete paracrine factors that initiate tissue repair processes, accelerate wound closure, and foster skin regeneration. Additionally, ASCs have demonstrated a high potential for skin regeneration due to their high abundance. Chan et al*.* demonstrated the feasibility of isolating ASCs even in patients with extensive burn injuries by utilizing the adipose layer of discarded burn skin [58]. The ASCs created discrete tubular networks and eventually dense and connected vascular structureswhen seeded inside the PEGylated-fibrin layer of a bilayered gel [58]. ASCs have been shown to promote vascular  maturation through their differentiation into ECs and perivascular cells [59]. Their autologous collection and ease of use make them attractive candidates for tissue engineering applications, facilitating enhanced vascularization and wound closure, with a high potential for expansion *in vitro* [60]. Additionally, Duttenhoefer et al*.* used 3D polyurethane as a biomaterial including hydroxyapatite nanoparticles to construct an *in vitro* prevascularized scaffold. The 9 mm^3^ scaffolds were built using 7 × 10^4^ EPC and 7 × 10^4^ ASCs in combination. Tubular structures began forming within the scaffolds as early as day 7 of culture, highlighting their potential for promoting  rapid vascularization [61].

Furthermore, Laschke and colleagues reported a massive angiogenic host tissue response after transplantation of porous polyurethane scaffold prevascularized by seeding ASCs spheroids [62]. They reported that approximately 40% of developed functional microvessels in the center of spheroid-seeded scaffold originated from GFP-labelled ASCs, which inosculated with ingrowing host vasculature [62]. Altman et al*.* demonstrated enhanced wound healing and ASCs differentiation into fibrovascular, endothelial, and epithelial components because of cells seeded on silk fibroin (SF)-chitosan (CS) scaffold [63]. Moreover, *.* Debski et al, confirmed the vascularization potential of ASCs seeded in prefabricated scaffolds, which showed ten-fold higher vessel densities in immunohistochemistry measurements as compared to controls [64]. The authors also reported that vasculogenesis of a scaffold can be improved if stem cells are injected in proximity of an area containing large nutritive vessels [64].

Several researchers have explored the composition of human adipose tissue and characterized the phenotypes of its constituent cells to assess their suitability for vascular tissue engineering. Previous reports have demonstrated that human adipose tissue-derived microvascular ECs (HAMECs) exhibit characteristic traits resembling ECs concerning morphology, molecular profile, and functional properties. Further, it was shown  that endothelial differentiation of ASCs alters their proteome, yet remains distinct from primary ECs and HAMECs, indicating a perivascular phenotype [65]. Despite this, ASCs were shown to differentiate into pericytes *in vitro* and stabilize HAMECs in nascent vessels, thereby contributing to HAMECs survival and vessel maturation. Thus, ASCs are pivotal for vascular tissue engineering due to their capability to remodel the extracellular matrix (ECM) and act as mural cells. The findings suggest that ASCs may play a key role in stabilizing and maturing the endothelium, partly by facilitating the assembly of its basement membrane [65].

Moreover, there are studies demonstrating differentiation of ASCs into vascular ECs to finally integrate them into the vascular network [66]. However, this is only possible through co-culturing ASCs with various specific ECs (such as HUVECs, human cardiac tissue ECs, and human pulmonary artery ECs) under specific culture conditions by utilizing a hydrogel constructs and endothelial growth mediumd supplemented with factors including epidermal growth factor (EGF), VEGF, insulin-like GF 1, basic fibroblast growth factor (bFGF), fetal bovine, and antibiotics, these cells can effectively differentiate into vascular ECs and/or contribute to the formation of a stable vascular network [66]. Further, ASCs have been shown to facilitate the recruitment of EPCs and augment their vasculogenic capabilities [67]. This collaborative interaction expedites the development of vascularized skin tissue, thereby enhancing the accessibility of nutrients, cytokines, and other molecular factors crucial for the skin healing process at the wound site. Notably, the synergistic effect of the implanted EPCs/ASCs co-culture system surpasses the individual effects of ASCs alone. The co-culture approach significantly advances skin tissue formation, primarily attributed to enhanced vascularization, which fosters enhanced recruitment of skin progenitor cells and facilitates the orchestrated action of cytokines involved in skin healing [67].

Thus, ASCs hold potential for regenerative medicine, but challenges exist for their clinical use, particularly in regards to their ability to promote blood vessel growth. The limitations include optimizing their isolation procedures and culture, understanding the mechanisms of their differentiation into blood vessel cells, and optimizing their therapeutic efficacy. Further research, particularly in animal models, is needed to address these challenges and advance ADSC-based therapies for clinical use.

### Pro-angiogenic GFs

The introduction of pro-angiogenic cytokines including VEGF, bFGF, and PDGF to the scaffolds is a traditional strategy for enhancing vascularization. To improve capillary development in cutaneous wound healing models, various scaffold types have been combined with such cytokines. These methods, however, frequently for recurrent administration or release-control mechanisms. However, the rapid degradation and diffusion of these cytokines still remain the major issues. According to Horikoshi-Ishihara et al*.*, the administration of a sustained-release VEGF or bFGF can trigger microvasculature formation and improve the vascularization of implanted 3D tissues [68]. Therefore, vascularization within engineered skin substitutes can benefit from the controlled release of GFs [68].

Several methods applying GFs have been already evaluated for enhancing vascularization of skin substitutes. As these methods frequently require repeated administration, skin substitutes implanted with cells that continuously produce GFs offer a viable alternative. For instance, seeding fibroblasts or keratinocytes causes faster wound vascularization because both cell types secrete distinct GFs. The integration of angiogenic GFs into prefabricated scaffolds or onto the surface of disintegrating beads for slow release to increase tissue angiogenesis directly after implantation are examples of such applications [68]. The addition of angiogenic GFs such as VEGF-A, FGF-2, and platelet-derived GF BB (PDGF BB) accelerated the process of angiogenic sprouting [68]. Although this GF-stimulated angiogenic response is rather short-term, it has the advantage of enhancing skin substitute survival by accelerating capillary formation and blood flow. Any expanding capillary network connected to a bioengineered skin substitute will constantly undergo remodeling to support the metabolic needs of the tissue [68].

Previous studies have demonstrated that keratinocytes overexpressing VEGF promote wound vascularization [69]. Furthermore, we have demonstrated previously that employing the prevascularized skin substitutes results in increased collagen type I deposition, increased dermal and epidermal cell proliferation, and decreased expression of wound healing markers [4, 5, 7–10]. Several cell types may promote vascular network development in skin substitutes through angiogenesis, vasculogenesis, or both. An ideal cell candidate for vascularization should meet the following criteria: (i) easyto harvest with low risk to the donor; (ii) rapidly expandable *in vitro *to amounts sufficient for vascularization of large skin substitutes; (iii) present no risk of malignant transformation; and (iv) non-immunogenic. Further, pro-angiogenicfactors such the VEGF, TGF-β, angiopoietin, or fibroblast GF (FGF) need to be well balanced when incorporated into transplants 70].

## Advanced vascularization strategies

### 3D bioprinting

The implementation of additive manufacturing in the field of biomedicine is known as 3D bioprinting. This technique has become a powerful and cost-effective method in tissue engineering and regenerative medicine, particularly when compared to traditional manual manufacturing methods. Many 3D bioprinters offer high precision and resolution, allowing for the creation of complex and finely detailed tissue structures in a relatively short time making the technology easily accessible for research institutions and medical facilities. Therefore, 3D bioprinting can significantly reduce the waiting time for tissue and organ transplants, potentially saving lives by providing timely replacements for damaged organs. As the technology becomes widely adopted, it has the potential to become more affordable, making it accessible to a broader range of research and healthcare facilities [71]. Four categories of 3D bioprinting can be distinguished based on the processes of fabrication: extrusion, inkjet printing, laser-induced forward transfer, and vat polymerization. 3D bioprinting is a cutting-edge technology that has gained significant attention in the field of tissue engineering. It involves the precise deposition of biological materials, such as cells and bioinks, layer by layer, to create complex three-dimensional structures.

#### Extrusion-based bioprinting (EBB)

Extrusion-based bioprinting (EBB) represents the most widely used bioprinting technique. In particular, coaxial bioprinting, a type of EBB, enables the fabrication of concentric biomaterial layers with cells and thus, can mimic crucial aspects of native tissues [72]. EBB is performed by loading specific bioinks into cartridges, which are then subsequently extruded onto a surface through a nozzle via either pneumatic pressure or mechanical forces. In general, three distinct methods are used for EBB: pneumatic-based extrusion, screw-based extrusion, and piston-based extrusion [72].

**Fig. 7:**
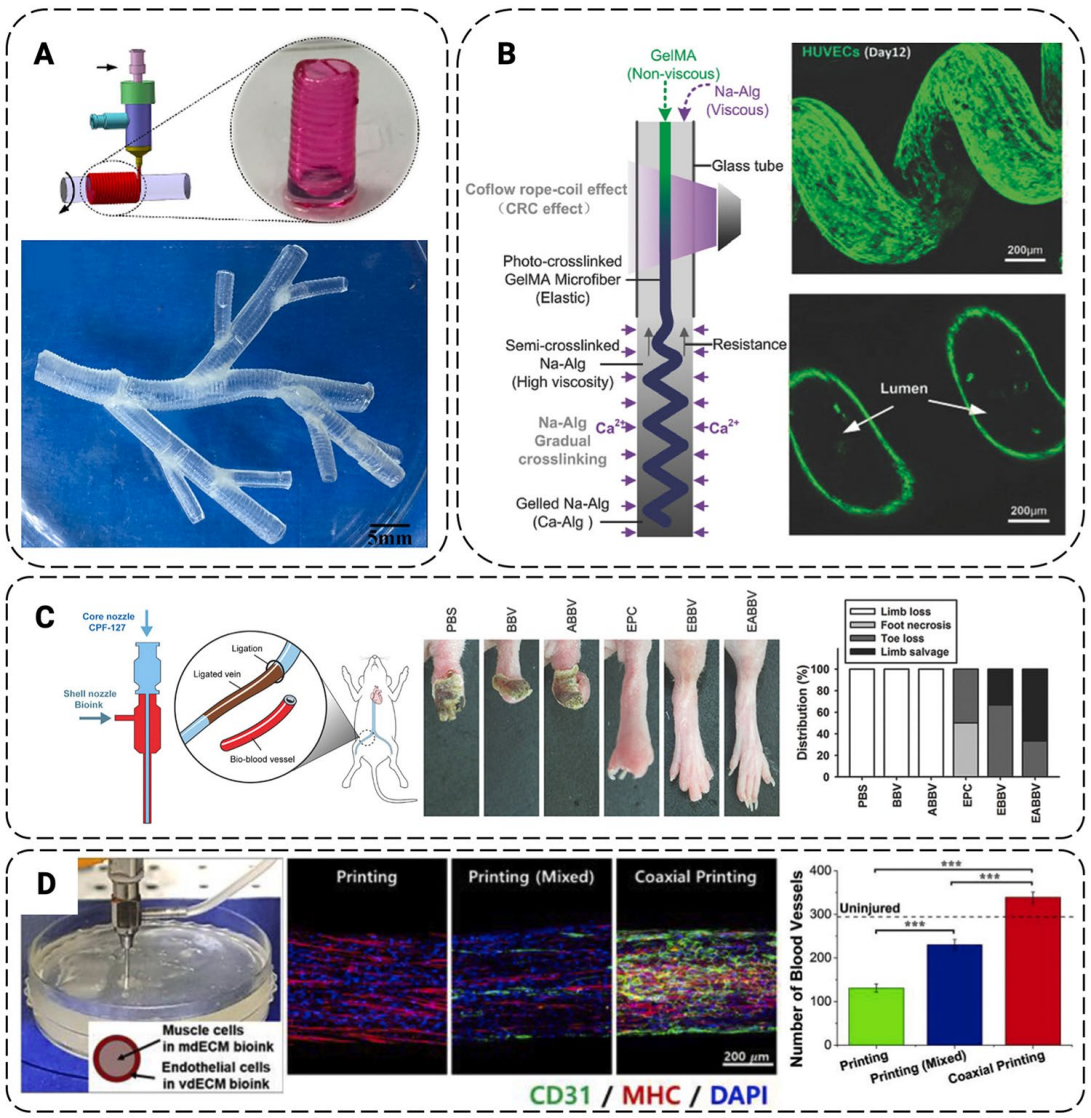
Applications of coaxial printing in vasculature engineering: **A** Vessels were fabricated by extruding sodium alginate onto a rotating rod (upper), followed by assembly into multiscale vasculature (lower). **B** Coiled-rope structures (upper right) were formed within a glass tube by crosslinking and adjusting the viscosity of the core and sheath fluids (left), providing space for endothelial cell lumen formation (lower right). **C** Artificial bio-blood vessels (BBV) were designed using a sacrificial core fluid (left), showcasing enhanced limb salvage in a mouse model when loaded with cells (EBBV) and a statin drug (EABBV) (center right). **D** Coaxially printed vessels (left) demonstrated cell differentiation* in vitro* (center) compared to single fluid printing (right) in a rat model. Scale bars: **A** 5 mm; **B** 200 μm; **D** 200 μm [Modified after 72]

In the last decade, coaxial bioprinting contributed significantly to the further development of tissue-engineered constructs with vascular networks. This type of bioprinting can be applied to control concentric multi-material deposition or improve resolution through inline crosslinking. Distinct combinations of hydrogels, cell-laden materials, or crosslinkers can be applied for the creation of vascular tubular structures, composite 3D structures, and complex multilayered structures. For example, perfusion of decellularized ECM-based proteins, followed by a low-viscosity cell-laden hydrogel was designed to increase cell attachment and migration. The first bioinks to be utilized in coaxial bioprinting were alginate and collagen, a mixture of which resulted in constructs that had the benefits of both collagen and alginate, namely soft material allowing cell interaction and aggregation and strong mechanical properties, respectively. In particular, the creation of vasculature is one of the major applications for coaxial bioprinting (Fig. 7). Importantly, coaxially-bioprinted constructs can mimic the key characteristics of the circulatory system. Currently, vascular structures including arteries and veins, which are 10–300 mm in diameter, as well as arterioles can be generated by coaxial printing [72].

However, one of the main challenges of coaxial printing remains the inclusion of cells since small nozzle sizes result in higher shear stress and damage to cells. In contrast, large nozzle sizes present an opposite as the bioink yield stress may be overcome by gravity, making printing actuation impossible. Thus, careful choice of nozzle dimensions and flow may enable a physiologically relevant scaffold organization.

#### Microfluidic technologies for 3D vascular systems

Microfluidics can facilitate the formation of a perfusable and functional 3D microvasculature in different organ-on-a-chip systems. Therefore, microfluidic technologies have emerged as useful tools, which can offer precise control over various aspects of the cellular microenvironment such as a different profile of fluid flow, gradient of various GFs, and mechanical properties of versatile biomaterials (e.g. stiffness, orientation, etc.). Furthermore, microfluidic technologies hold great potential to model pathological conditions to study vascular-related diseases [73].

Importantly, microfluidics can mimic physiological 3D microstructure of blood vessels (i.e., circular cross-section) and blood vessel polarization from the apical (luminal) to basal (abluminal) axis, which is important for directed secretion of proteins involved in the cell shape to form a lumen [73]. Besides that, microfluidics also enables the continuous perfusion of cell culture medium to supply oxygen and nutrients as well as the removal of waste products, which is crucial to maintaining the long-term survival of vascularized microtissues. With the combination of various biomaterials and tissue engineering techniques, three main types of 3D *in vitro* microfluidic vascularization strategies have been developed: (1) EC lining-based methods, (2) vasculogenesis and angiogenesis-based methods, and (3) hybrid methods.

**Fig. 8:**
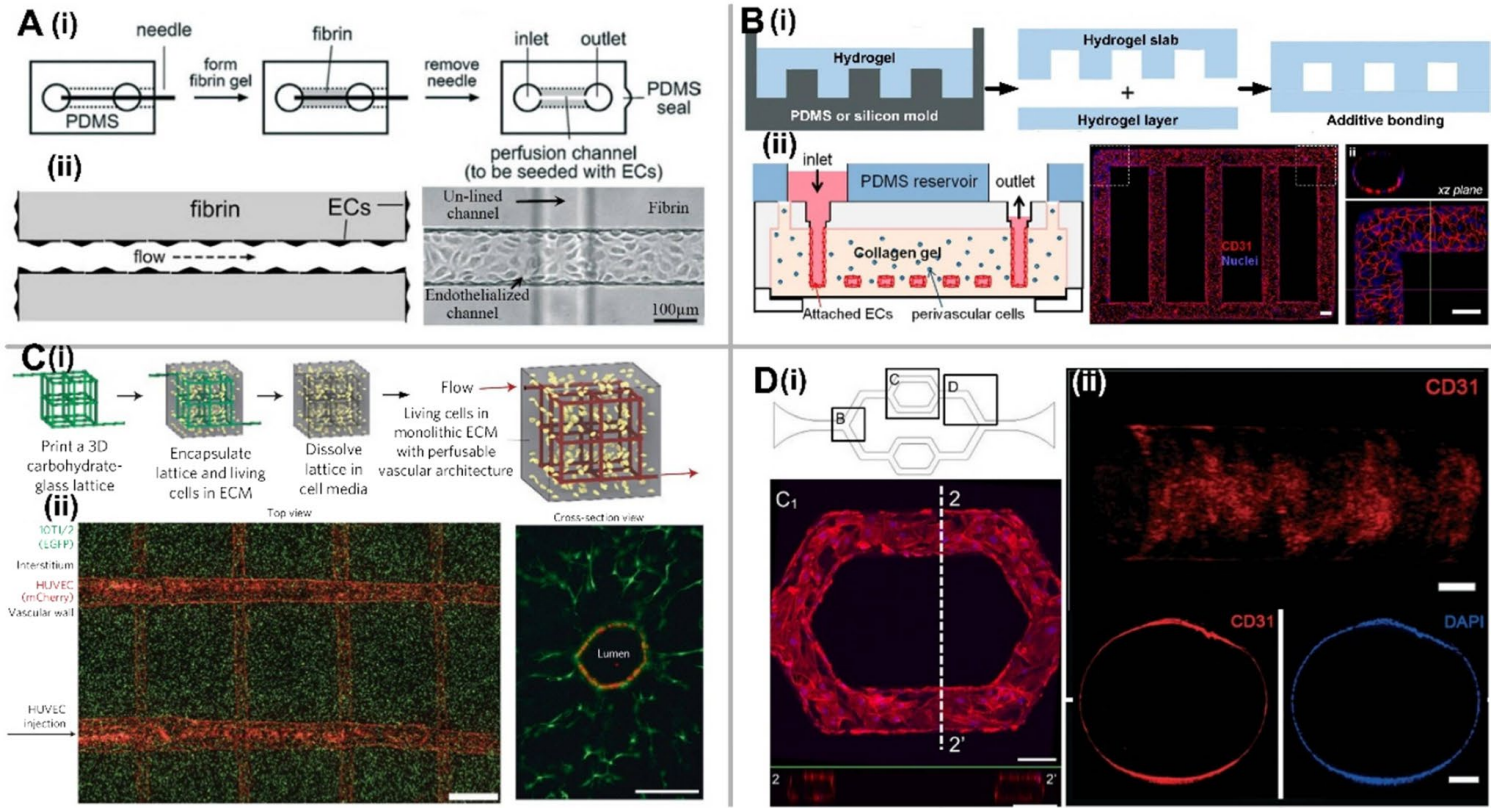
Vascularization strategies using ECs *in vitro*: **A** Microneedle-based Removable Method: (i) A needle-molding technique to form fluidic channels within hydrogels. (ii) Endothelialized microchannels under the influence of fluid flow. **B** Micropatterned Planar Hydrogel Slab Bonding Method: (i) Fluidic hydrogels created via micromolding from PDMS or silicon molds, followed by bonding. (ii) Micrographs exhibit the shape of microvessel networks within collagen gel constructed from a micropatterned silicon stamp, along with confocal sections of endothelialized microfluidic vessels immunostained with CD31. Scale bar, 100 μm. **C** Dissolvable Materials-Based Sacrificial Micromolding Method: (i) A schematic showcases a 3D interconnected microvessel network formed by casting a carbohydrate glass lattice as the sacrificial element with a 3D printer. (ii) Micrographs reveal HUVECs expressing mCherry attached to the hydrogel wall for generating the microvessel network and an endothelial monolayer-lined vascular lumen surrounded by 10T1/2 cells after 9 days in culture. Scale bars, 1 mm and 200 μm. **D** EC Lining Inside a PDMS-Based Microfluidic Channel: (i) A schematic depicts a PDMS-based microfluidic channel, alongside a confocal image of endothelial cells within the channel. (ii) Confocal reconstruction images show the complete lumen formed by HUVECs inside the PDMS tube, along with fluorescence micrographs of cross-sectional views of an endothelialized PDMS tube stained with CD31/nuclei. Scale bars, 100 μm (top) or 200 μm (bottom) [modified after 73]

The EC-lining method represents one of the first established techniques, which has been broadly used. It is achieved bycreating a microfluidic channel and lining it with EC to form a monolayer on the inner walls of the microchanneles (Fig. 8).

The major advantage of the EC-lining method is that the vascular geometry and dimensions can be easily controlled. Besides, shear stress can be precisely calculated based on the channel dimensions and the applied flow rate. The hollow single channel or a channel network can be made of either hydrogel or polydimethylsiloxane (PDMS). For hydrogel-based microchannel, various micro-molding methods reported in the literature so far can be grouped into three main types: (1) microneedle-based removable method for a single microchannel, (2) micropatterned, planar hydrogel method for single-layer microchannel network, and (3) dissolvable material-based sacrificial micro-molding method for multi-layer microchannel network. Since cells might not adhere tightly to the PDMS surface inside a PDMS-based microfluidic channel, a thin layer of basement membrane ECM proteins (e.g., laminin, fibronectin, collagen IV, etc.) need to be coated onto the microchannel inner walls to enhance cell adherence. However, the main drawback of EC-lining methods is the fact that they cannot mimic physiological vascular formation *in vivo* [73]. Indeed, this can be only reached using vasculogenesis and angiogenesis-based methods.

**Fig. 9:**
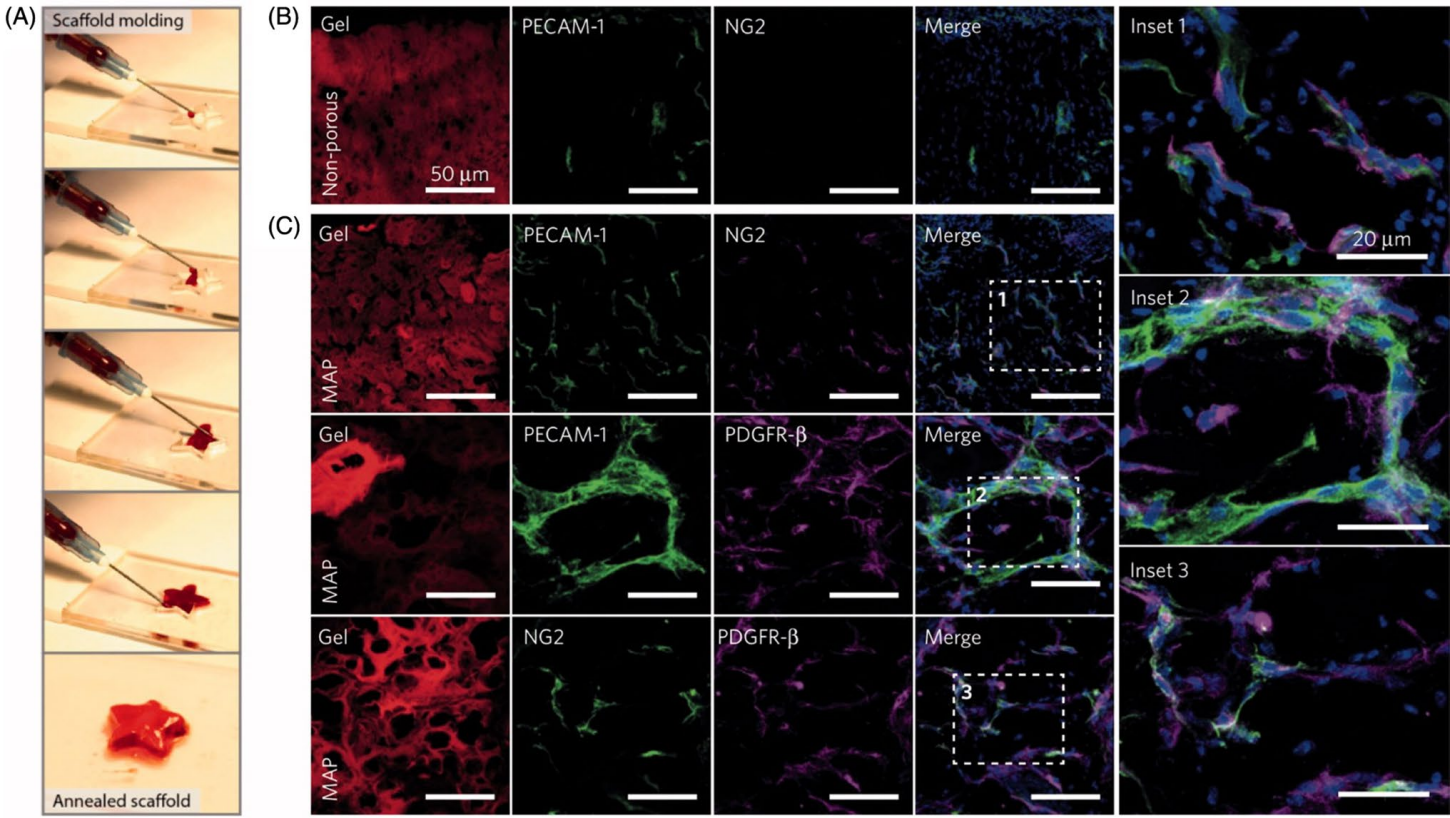
Development of MAP gel using microfluidics. **A** Application of MAP gel to produce any kind of 3D shape through 25-gauge syringe. **B–C** Detection of vasculature by staining for endothelial cell marker PECAM-1 in MAP scaffolds, 5 days after transplantation onto mice injured skin [modified after 76]

Several studies highlight the diverse approaches employed to enhance vascularization in skin tissue engineering. Skardal et al. demonstrated an innovative approach to enhance the vascularization of skin defects in a mouse wound model. They utilized bioprinted amniotic fluid-derived stem cells embedded within fibrin-collagen hydrogels [74]. This combination effectively promoted the formation of new blood vessels in the injured skin tissue [74]. In another study, Michael et al. employed laser-assisted bioprinting to create artificial skin by implanting keratinocytes and fibroblastsin Matriderm®, a dermal template [75]. Upon implantation of these constructs into mouse dorsal skinfold chambers, the authors observed the development of new blood vessels originating from the surrounding host tissue. This vascularization process was mainly stimulated by the secretion of vascular endothelial GF (VEGF) by the co-implanted keratinocytes [75]. Additionally, researchers utilized a microfluidic water-in-oil emulsion technique to produce microporous annealed particle (MAP) gels, which are injectable materials for *in situ* skin regeneration (Fig. 9A). These scaffolds facilitated accelerated skin regeneration and the ingrowth of microvasculature* in vivo* [76]. Importantly, the vascularization process was characterized by early pericyte stabilization of the newly formed microvessels within the first seven days (Fig. 9B, 9C), indicating a crucial role in vascular maturation and stability [76].

One important concept in vessel network formation is the establishment of a vascular hierarchy, where vessels of different sizes and functions are organized hierarchically. This hierarchical organization is critical for efficient blood flow distribution and tissue perfusion. Arterioles, capillaries, and venules have distinct structural and functional characteristics, and their formation involves spatially and temporally coordinated interactions between ECs, mural cells, and perivascular cells. Studies have elucidated various mechanisms involved in the formation of hierarchical vessel networks. For example, recent research has highlighted the role of EC heterogeneity, differential expression of angiogenic factors, and dynamic cell–cell interactions in shaping vessel hierarchy. Additionally, emerging evidence suggests that mechanical forces, such as blood flow patterns and tissue stiffness, contribute to the spatial organization and remodeling of vessel networks.

In human skin, the capillary network consists of both blood and lymphatic capillaries present through the entire dermis. Interestingly, the upper papillary and lower reticular dermal parts differ with respect to vascularization pattern. There are two primary horizontal plexuses composed of arterioles and veins in the dermal compartment, namely the superficial sub-papillary plexus (SSP) and the deeper cutaneous plexus (DCP). Arterioles that supply blood to the muscles and the hypodermal layer form the DCP, which reside between the subcutaneous and the cutaneous compartment. The arterioles and veins are arrange in a vertical vascular pattern to connect to the SSP, which is located between the epidermis and the papillary dermal compartment. The vessels in the DCP differ in morphology from those in the SSP. DCP vessels have a wider diameter (10–35 µm in the SSP and 40–50 µm in the DCP) and thicker walls. Individual arterioles in the dermal papilla form separate capillary vessel loops that have an ascending limb, an intra-papillary loop, and a descending limb that fuses with post-capillary venules. The capillary loop supplies blood to a skin area that is between 0.04 and 0.27 mm^2^ in size. Numerous anastomoses in the skin vasculature through which blood flows play an important role in temperature regulation. So far the complex vascular plexus of human skin could not be reproduced *in vitro* [77].

Recently, researchers have presented a novel approach for constructing a hierarchical vessel network using induced spontaneous anastomosis in a tumor model-on-a-chip. They developed a microfluidic platform that enables the formation of hierarchical vessel networks to support tumor growth. Fabrication of a microfluidic device, consisting of multiple interconnected channels, was designed to mimic the hierarchical organization of blood vessels *in vivo*. Next, ECs were seeded within the channels and spontaneous anastomosis, a process where adjacent vessels fuse to form interconnected networks, was induced. This approach allowed the generation of capillary-sized vessels, arteriole-sized vessels, and venule-sized vessels within the microfluidic device, resembling the complexity of native vascular networks [78].

There are a few important issues to think about before building a new *in vitro* microvasculature or modifying an existing one depending on size and flow characteristics. First, any microfluidic system containing cultivated cells is severely damaged by air bubbles, but this can be avoided with the right approach (priming tubing and connecting fluidic ports in a bubble of liquid). Second, it is difficult to evenly distribute a high density of ECs inside the microfluidic channels with small diameters due to the blockage, thus EC lining methods are only suitable to construct large blood vessels with diameter greater than 50 μm. The time required for device design and development is another factor to take into account; it can take anywhere from days for PDMS devices to weeks for certain gel devices, however, a cutting-edge platelet-rich plasma-culturing method may significantly accelerate this procedure. The choice of assays that can be successfully carried out using *in vitro* microvascular systems is the final important factor to take into account. Many of these assays may require live-cell microscopy imaging, keeping in mind that microvascular failure might manifest as bleeding, thrombosis, remodeling, or altered perfusion. Whereas immunofluorescent labeling of specific antigens and adhesion molecules represents an easy method, single-cell-based approaches linked to proteomics and lipidomics may require the pooling of many devices to obtain the necessary numbers of cells for analysis [79].

In conclusion, 3D bioprinting is an innovative technology with immense potential to revolutionize medicine and healthcare. While it offers numerous advantages, including tissue customization and complex structure creation, it also faces technical, cost, and regulatory challenges that must be addressed to unlock its full potential and ensure its safe and effective use in clinical practice. Continued research and development in the field will likely lead to further advancements and overcome many of the current limitations.

#### Biomaterials used for 3D bioprinting

##### GelMA

GelMA, a gelatin derivative with a significant number of methacrylamide groups and fewer methacrylate groups, is another type of hydrogel with several biomedical applications due to its versatile physical attributes and compatible biological properties. Several authors used multiple terms for GelMA, such as gelatin methacrylamide, methacrylated gelatin, methacrylamide modified gelatin, or gelatin methacrylate (GelMA). Because GelMA hydrogels contain peptide motifs that bind to cells and activate matrix metalloproteinase (MMP), they bear a striking resemblance to some of the fundamental characteristics of native ECM. These peptides facilitate the growth and division of cells within GelMA-based scaffolds [80].

To create covalently crosslinked hydrogels, GelMA is exposed to UV light in the presence of a photoinitiator, a process known as photoinitiated radical polymerization. Gelatin is a byproduct of the hydrolysis of collagen, which is the main component of ECM in most tissues. It contains numerous arginine-glycine-aspartic acid (RGD) sequences that facilitate cell attachment, as well as matrix MMP target sequences that are appropriate for cell remodeling. Since its first synthesis, GelMA hydrogels have been thoroughly studied in terms of physical and biological properties. GelMA applications range from tissue engineering to medication and gene delivery. Various forms of GelMA have been utilized so far, including 3D hydrogel, electrospun fibrous membranes, and 3D printed scaffolds. These scaffolds may fulfill the needs of skin engineering repairs, tendon, bone, cartilage, vasculature, etc. when combined with other polymers, GFs, and small molecule drugs [80]. Moreover, GelMA is a potential raw material for organ-on-chip due to its high-performance biofabrication and biocompatibility. Moreover, modified GelMA can be used for the fabrication of food analysis tools [80].

GelMA scaffolds have been demonstrated by Zhao et al*.* to facilitate the multi-layered epidermis development with high keratinocyte proliferation [81]. To further enhance wound healing, they also constructed a 3D, completely cellularized scaffold that mimics the natural dermal ECM using GelMA hydrogels. In addition, Zhao et al*.* applied GelMA in a rat full-thickness skin wound healing model to increase vascularization for the treatment of random skin flap distal necrosis [81]. Furthermore, Zhou et al*.* developed GelMA-based intelligent, responsive wound dressing vesicle systems that give fluorometric/colorimetric response (green) to bacterial infection on the wound site and release an encapsulated anti-microbial agent of vesicles to either block or kill pathogenic bacteria, including *P. aeruginosa* and *S. aureus*, while providing a visual infection alert [82]. This approach aims to mitigate antibiotic resistance and the excessive use of antimicrobials while extending the efficacy and stability of the encapsulated antimicrobial agents. This is achieved by ensuring the controlled release of antimicrobials specifically triggered in response to pathogenic bacteria. A recent contribution by Jahan et al*.* demonstrated that Ag-nanoparticle entrapped GelMA scaffolds improve wound healing, particularly for deep skin wounds^83^ . When employed as a wound dressing, these scaffolds trigger fibroblast migration and reduce microbial infections [83].

Moreover, GelMA has a wide range of applications to facilitate 3D vascular network formation because of its adaptable mechanical characteristics. For example, Chen et al. demonstrated that GelMA hydrogels may promote the development of human progenitor cells-based vascular networks [84]. Additionally, they demonstrated that the degree of methacrylation affects the malleable physical properties of GelMA modulating the extent of vascular development [84]. In particular, GelMA with 49.8% methacrylation creates a  softer hydrogel than GelMA with 73.2% methacrylation [84], with the latter showing enhanced vascular development. GelMA’s vascular formation performance can be further improved by combining it with drug release systems. For instance, Chen et al*.* created a GelMA hydrogel to deliver desferrioxamine through a continuous and steady release [85]. This hydrogel network promotes the expression of HIF-1α, an important activator of vessel formation, and thus creates a favourable environment for EC proliferation and migration [85]. Further, using an extrusion technique, Wang et al*.* created a living photosynthetic scaffold made of microalgae, alginate, and GelMA that resembles the properties of tissues or organs [86]. As the inner and outer phases, respectively, they employed a solution of microalgae, alginate, and GelMA and gelatin fluid containing calcium chloride. The hollow fibers were created by the cross-linking of alginate and Ca^2+^, and the GelMA component was then photopolymerized by UV light. Moreover, it was shown that the resulting hydrogel fibers may be piled on tissue *in situ* to create a 3D scaffold, suggesting that this technique could be a novel and interesting approach for 3D bioprinting [86].

Despite great advancements in 3D bioprinting, printing multi-layered tubular tissues, like arteries, is still challenging. In this respect, GelMA represents a promising biomaterial for vascular regeneration. GelMA has been synthesized in various forms including hydrogels, 3D bioprinted scaffolds, and electrospun fibers, thanks to its controllable physical properties. Pi et al*.* demonstrated that multi-layered tubular tissues may be bioprinted in a single step using the multichannel coaxial extrusion system using GelMA, alginate, and 8-arm polyacrylate as the bioink [87]. Meanwhile, a favorable environment for cell adhesion and proliferation may be provided by GelMA hydrogel, which shows strong hydrophilicity and naturally occurring cell-binding motifs. Apart from 3D printing, there are other uses for the combination of GelMA and electrospinning in vascular tissue engineering. For example, Hassanzadeh et al*.* used a chitin nanofibrous system to create a self-assembly GelMA hydrogel, resulting in the formation of hybrid films with varying chitin nanofiber content [88]. The straightforward self-assembly procedure of these hydrogels is compatible with soft lithography techniques for microstructure fabrication. Importantly, HUVECs co-cultured with human MSCs demonstrated high proliferation and alignment on the micropatterned hydrogels. Furthermore, those matrices showed expression of vasculogenic markers, confirming cell differentiation and the establishment of stable vasculature in these substrates. [88].

Altogether, hydrogels like GelMA, silicone, and ECM like collagen type I are some of the most frequently applied biomaterials that are used in 3D bioprinting techniques. However, these biomaterials have also some drawbacks, including the need for the material to be liquid before printing, the requirement for quick molding after printing, insufficient physical-mechanical properties, channel collapse, inconsistent biological drop and tissue regeneration, high oil-liquid interfacial tension, and other restrictions. The process of extruding bioink or cell-laden materials through a nozzle or printhead can subject the printed cells to shear stress. Excessive shear stress can damage or disrupt delicate biological structures and compromise the viability and functionality of the printed cells. Printhead clogging is a common issue in 3D bioprinting systems. The bioinks used in the process can contain particulate matter, cells, or biomaterials that may clog the printhead or nozzle. This can lead to interruptions in the printing process, affecting the quality of the printed tissue or organ. The diameter of the printing nozzle or printhead can impose limitations on the size and resolution of the printed structures. Smaller nozzle diameters may offer higher resolution in coaxial bioprinting but can limit the speed of printing, while larger nozzles may enable faster printing but with reduced precision. Balancing these factors can be challenging, especially for complex tissue engineering applications [89].

Biomaterial models are designed and manufactured layer by layer using techniques like photopolymerization and microfluidics to manipulate the bioink. As a result, the bioinks used to produce the biomaterials mostly define their biocompatibility. The development of highly organized microvascular networks with distinct branching and hierarchical patterns is made possible by two fast-evolving technologies: microfluidics and 3D bioprinting. On the one hand, phase-changing hydrogels, soluble factors, and endothelial and parenchymal cells can be co-assembled using 3D bioprinting in a high-throughput and reliable manner. Implementation of the proper bioreactor perfusion methods enables ECs to be uniformly seeded into the microfluidic channels of 3D hydrogels. Skin tissue engineering is recently beginning to employ these approaches, although these techniques have been extensively studied in the fundamental angiogenesis and vascular research field.

##### Decellularized ECM

Conventional skin substitutes often fall short of recapitulating the intricate architecture and multifaceted functionality of native dermal tissues. Therefore the emergence of decellularized extracellular matrix (dECM) generated after the removal of cellular components, holds promise in circumventing these challenges. dECM, derived from endogenous ECM, closely mimics its structural and compositional properties, thereby fostering a conducive microenvironment for cellular regeneration. Additionally, dECM exhibits favorable bioactivity, minimal immunogenicity, and abundant availability, rendering it a promising biomaterial for the restoration and rejuvenation of cutaneous tissue. Recent research has highlighted the pivotal role of dECM in the intricate process of skin wound repair. Characterized by its abundance of biomolecules and unique 3D structure conducive to cellular behavior, dECM stands as an optimal bioactive scaffold for skin repair and regeneration. The fibrous network architecture of dECM fosters an appropriate microenvironment for cellular proliferation, while its bioactive constituents modulate crucial cellular functions such as adhesion, migration, proliferation, and immune regulation during wound healing.

The inception of decellularization dates back to 1948, and since then, the advancement of decellularization technologies has been substantial. For example, in the 1970s, the isolation of basement membrane was achieved, while the production of acellular small intestinal submucosa matrices began in 1995. Subsequently, decellularized whole heart, lung, and kidney scaffolds were developed in 2008, 2010, and 2013, respectively. Simultaneously, evaluation methodologies were further developed; the establishment of ECM’s matrisome occurred in 2012, followed by proteomic, and glycosaminoglycanomic analyses in 2020 and 2021, respectively. The development of a commercial acellular dermal matrix in 1995 allowedthe firsthuman application in 1996. Since then, a plethora of dECM and dECM-based materials have been synthesized and utilized for skin regeneration. A recent study by Jiang et al. revealed that dECM derived from skin tissue exhibits superior physical stability and biological composition compared to conventional collagen bioink [90].

**Fig. 10:**
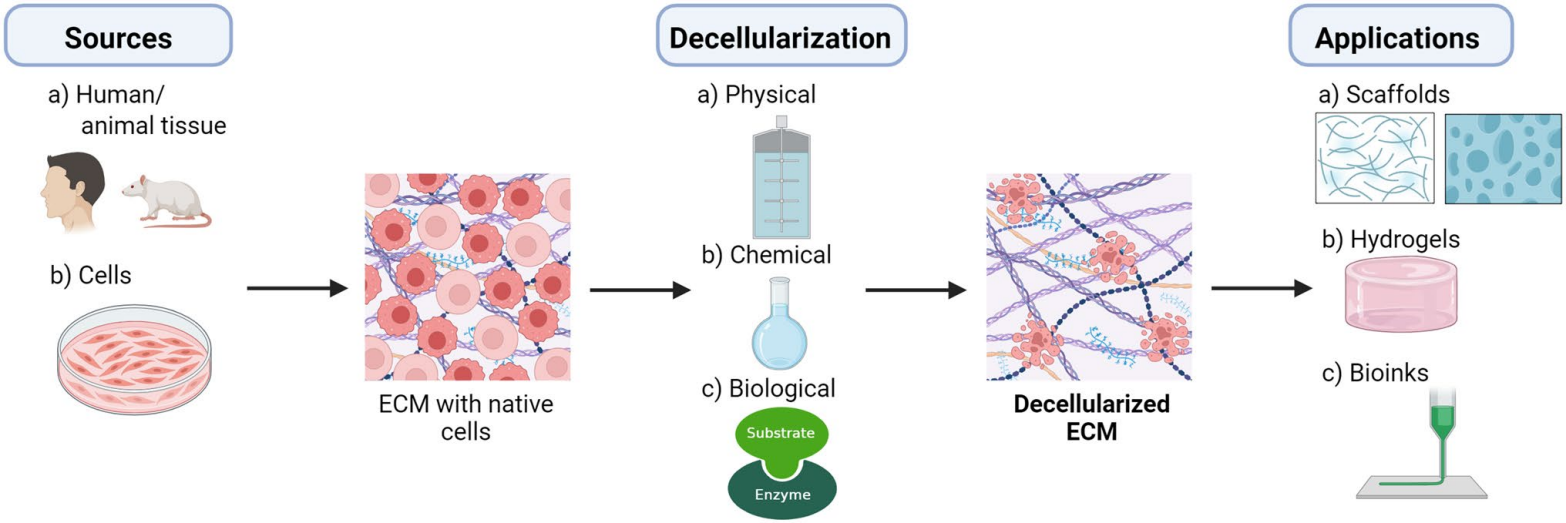
Schematic diagram for preparation and application of dECM (prepared with BioRender)

The ECM serves as a bioactive substrate rich in biochemical cues essential for cellular events crucial in wound healing. Nonetheless, the presence of cellular and nuclear remnants within the ECM may induce cytotoxic effects *in vitro* and trigger immune responses afterwards *in vivo*, potentially compromising the wound repair and thus, therapeutic outcomes. Consequently, a meticulous decellularization protocol for ECM-derived materials becomes imperative before application, with the primary objective of minimizing immunogenic components. Physical techniques, such as freeze-thaw cycles, mechanical agitation, pressure application, sonication, and supercritical CO_2_ treatment, represent straightforward approaches to ECM decellularization (Fig. 10). Notably, their avoidance of chemical reagents renders them particularly favored among researchers. Deng et al. [91] employed freeze-thaw cycling methods to produce ECM-PLGA materials, with subsequent DNA electrophoresis and quantitative analysis indicating significant removal of DNA components. Moreover, various chemical agents, encompassing acids, bases, detergents, and hypo/hypertonic solutions, have demonstrated notable efficacy in removing nuclear and cytoplasmic components. In investigation performed by Ng et al., Triton X-100 and NH_4_OH solution were utilized to decellularize human umbilical cord-derived mesenchymal stem cells, yielding favorable results [92, 93]. Unlike physical decellularization methods, theis method using chemicals offer efficient removal of potentially immunogenic ECM components within a short timeframe, alongside with inherent sterilizing properties. However, excessive detergent concentrations and prolonged exposure to chemicals may lead to the depletion of vital GFs and disruption of ECM ultrastructure. Consequently, optimizing the duration and concentration of chemical agents is of paramount importance for achieving satisfactory decellularization outcomes. Furthermore, process-related impurities and chemical residuals within dECM poss a significant challenge, and may cause host inflammatory responses. In addition to physical and chemical methodologies, biological techniques are also available for the removal of cellular components. For example, nucleases exhibit notable efficiency in eliminating DNA and RNA contents, while lipases are utilized for specific lipid removal. However, akin to chemical approaches, biological methods often result in incomplete decellularization, residual reagents, disruption of ECM ultrastructure, and potential inflammatory reactions. For instance, prolonged exposure to trypsin may induce irreversible damage to ECM collagen components. Conversely, when employed in conjunction with the chelating agent ethylenediaminetetraacetic acid (EDTA), the risk of immune response activation can be mitigated.

dECM can be categorized into two distinct groups according to the source of the ECM: organ-/tissue-derived and cell-derived dECM (Fig. 10). The dECM sourced from organs/tissues and cells each possesses distinct attributes. Organ/tissue-derived dECM boasts an optimal yield and preserves its native 3D structure along with multidirectional active components, yet it carries inherent risks such as immunogenicity, cell permeability, and potential disease transmission. In contrast, cell-derived dECM inherits natural bioactive factors and proteins from donor cells, mitigating the risk of pathogens and immunogenic macromolecules compared to organ/tissue-derived dECM. However, cell-derived dECM lacks a three-dimensional structure, necessitating the incorporation of additional molecules or the combination with other scaffold materials to bolster mechanical strength in practical applications [94]. Concurrently, the conventional two-dimensional (2D) culture system falls short in meeting the demands of large-scale preparation, prompting the need for the development of three-dimensional (3D) culture systems to rapidly upscale cell quantity and enhance ECM production efficiency.

Thus far, a diverse array of organs/tissues has been employed in skin tissue engineering endeavors, encompassing skin tissues, perinatal-related tissues, adipose tissues, small intestinal submucosa, fish skin, heart, lung tissues, and beyond. Pigs represent the primary source among animal-derived dECM biomaterials due to their widespread availability and ample supply. Numerous investigations have highlighted the close resemblance in composition and functionality between porcine skin ECM and its human counterpart as compared to ECM from other animal sources. In a recent study by Liu et al. [95] proteomic analysis of porcine skin-derived dECM revealed elevated levels of angiogenesis-associated proteins, pointing towards its potential role in wound healing by regulating angiogenesis. Currently, porcine skin and small intestine submucosa rank as the most extensively utilized dECM tissues, both of which have transitioned into  commercial products for clinical applications. Human skin, adipose, and perinatal tissues represent the primary sources of dECM utilized in skin regeneration. Perinatal tissues, encompassing the placenta, umbilical cord, and amniotic membrane, fulfill various functions during fetal development but are typically discarded postpartum. Extensive research has underscored the rich trove of diverse GFs and cytokines present in perinatal-related tissues, including EGF, TGF-β, FGF, PDGF, and VEGF. These bioactive agents play pivotal roles in fibroblast migration, MSCs homing, re-epithelialization, and neovascularization, rendering them indispensable for wound healing and early-stage tissue repair and regeneration. In a recent study [92], the dECM extracted from human, pig, and rat skin underwent comprehensive proteomic and bioinformatics analyses. In comparison to human-derived dECM, both pig and rat skin-derived dECM showed a deficiency in proteins linked to inflammatory modulation and the innate immune response. Additionally, they exhibited markedly lower levels of most proteases and protease inhibitors. These findings suggest that human-derived dECM may establish a more favorable microenvironment for enhancing wound healing. Compared to organ/tissue-derived sources, cell-derived dECM presents a reduced presence of immunogenic components and a diminished potential for pathogen transmission. Furthermore, dECM can be obtained through the cultivation of the patient’s own cells, facilitating “autologous tissue engineering”. Notably, during *in vitro* ECM deposition, specific modifications of dECM can be achieved by altering culture conditions or introducing particular stimuli, a feat challenging to replicate in organ-/tissue-derived dECM. For instance, Dong et al. [96] successfully generated dECM with immunomodulatory properties by supplementing IFN-γ during culture. Similarly, the bioactivity of cell-derived dECM can be augmented through the co-culture of two or more cell types. Carvalho et al. [97] observed that dECM produced via the co-culture of bone marrow-derived MSCs (BMSCs) and HUVECs significantly enhanced the osteogenic differentiation and angiogenic potential of BMSCs *in vitro*. Moreover, cell-derived dECM can be tailored into various formats depending on the intended applications, including 2D layers and 3D structures. Cell-derived dECM has found widespread application in skin, nerve, bone regeneration, and other domains, yielding favorable tissue regeneration outcomes. In the context of skin regeneration, diverse fibroblast types serve as primary sources of dECM. As the predominant cell type in the dermis, fibroblasts possess robust ECM secretion capabilities, resulting in dECM primarily comprised of collagen, closely resembling native skin tissue in comparison to traditional biopolymers. Furthermore, various fibroblast subtypes exhibit distinct phenotypes, protein compositions, secretory profiles, and mechanical properties. Researchers have highlighted the morphological, functional, and compositional differences observed in ECM derived from three distinct fibroblast subtypes within the human skin dermis. These findings underscore the versatility of maximizing ECM bioactivity through diverse methodologies, suggesting that cells originating from the same source are not invariably essential.

To sum up, dECM encompasses a plethora of GFs and bioactive molecules crucial for regulating cell maturation, migration, proliferation, and other vital biological functions essential for skin repair processes. Following decellularization, dECM can be generated in various forms such as powders, gels, foam, and sheets. Moreover, it can be synergistically combined with cells, GFs, and other synthetic peptides to create advanced bioactive scaffolds. Over time, a myriad of dECM-based materials have been developed for skin repair and regeneration, spanning from porous scaffolds and fibrous scaffolds to hydrogels and bioinks.

**Porous scaffolds,** organ-/tissue-derived dECM, sourced from both prenatal and skin tissues, have found application in skin regeneration. Following meticulous decellularization, these dECM scaffolds retain a plethora of bioactive molecules and showcase a heterogenous morphology characterized by a high degree of interconnectivity. Researchers have devised a notably porous dermal substitute utilizing human placental dECM, demonstrating its capacity to expedite wound healing and foster skin regeneration through a bilayer structure comprising the epidermis and dermis [98]. Conversely, while cell-derived dECM boasts numerous advantages over its organ/tissue counterparts, its primary limitation lies in the absence of requisite mechanical strength and structural integrity for practical applications. An effective remedy entails depositing ECM directly onto existing scaffolds, thereby achieving an optimal amalgamation of biological activity and mechanical robustness. Developing hybrid scaffolds by integrating dECM with other natural macromolecules emerges as a promising strategy to enhance the mechanical characteristics of dECM. In a study, human lung fibroblast dECM (fdECM) was combined with type I porcine collagen to fabricate bioscaffolds tailored for skin wound healing and remodeling purposes [99]. Remarkable advancements in skin healing were observed, accompanied by restoration of epidermal barrier function and revitalization of collagen, hair follicles, and the subepidermal plexus in both full-thickness wound and diabetic ulcer models [99].

**Fibrous scaffolds**, characterized by nanofiber structures, are extensively employed in both hard and soft tissue engineering applications, offering an ideal microenvironment for cell adhesion, proliferation, and differentiation [100]. A straightforward approach for fabricating dECM fibrous scaffolds involves initially culturing cells on pre-existing scaffolds, followed by subsequent decellularization. In a notable study, human adipose-derived stem cells (ADSCs) were seeded onto  silk fibrin (SF) scaffolds produced via electrospinning [101]. After a 7-day culture period, these cell-loaded scaffolds underwent decellularization. Electron microscopy and immunofluorescence analyses revealed robust attachment and proliferation of ADSCs, along with the maintenance of their multipotent differentiation capabilities. Animal studies further demonstrated that the ADSC-dECM-SF scaffold effectively promoted skin healing in diabetic mice [101]. Furthermore, the fabrication of dECM fibrous scaffolds with bilayer architectures has garnered significant attention. Typically, biomaterials such as PVA, CS, and SF are applied as coatings on dECM to enhance their mechanical properties and mitigate degradation rates [102–105 Electrospun fibrous scaffolds have emerged as a focal point of interest in recent years, owing to their porous structures and high surface area, closely resembling those of the native ECM. Additionally, the incorporation of drugs and GFs directly into polymer solutions during the electrospinning process enables simultaneous functional and structural optimization [90].

**dECM-based hydrogels**, characterized by their thermosensitive and sol-gel transition properties, offer distinct advantages such as injectability and suitability for application in irregularly shaped defects, compared to membrane-formed scaffolds [106]. Enzyme digestion represents the most common method for preparing dECM hydrogels. In brief, harvested dECM undergoes lyophilization and grinding into powder, followed by digestion in acidic pepsin solutions and subsequent dissolution. The digestion process is halted by the addition of NaOH solution, yielding the dECM pregel solution [108]. [92, 107] pioneered the development of dECM hydrogels sourced from perinatal tissues and evaluated their impact on the wound healing process in mouse skin. Liquid chromatography-tandem mass spectrometry analysis unveiled distinct compositions among placental, umbilical, and amniotic membrane dECM hydrogels. Subsequent animal studies showcased the superior anti-inflammatory, angiogenic, and epithelial regeneration properties of placental dECM hydrogels [107]. Presently, stem cell-driven tissue engineering stands as a promising avenue for skin regeneration. Among the myriad of stem cell types, ADSCs stand out as particularly desirable due to their accessibility and capacity for multilineage differentiation [109]. Moreover, ADSCs have demonstrated efficacy in promoting skin regeneration by facilitating EC recruitment and secreting key bioactive factors such as VEGF and EGF. In a notable study, an injectable hydrogel was engineered by combining human adipose tissue dECM with methylcellulose (MC) for ADSC delivery [110]. Within the dECM-MC hydrogel, ADSCs maintained robust viability, and the ADSC-laden dECM-MC hydrogel exhibited promising outcomes in skin regeneration, fostering re-epithelialization and neovascularization. Nevertheless, devising an efficient strategy for delivering stem cells to wound sites via biomaterials remains a formidable challenge. Hydrogels offer a potential solution by facilitating *in situ* cell delivery through direct cell encapsulation^111^ . Notably, dECM hydrogels have emerged as a compelling option, as they furnish an optimal microenvironment conducive to the biological efficacy of stem cells [90].

**dECM bioinks** The fusion of 3D printing and biology in 3D bioprinting holds immense potential for fabricating various organs or tissues [112]. One of the primary hurdles in utilizing 3D bioprinting for skin regeneration lies in the application of an optimal bioink. This bioink must not only be printable but also possess exceptional biological properties, including the ability to facilitate cell attachment, diffusion, proliferation, and other physiological functions of printed cells [113]. Numerous investigations have highlighted the advantageous bioactivity of dECM-derived bioink, which can sustain cell viability, enhance cell proliferation, and foster cell differentiation, thereby facilitating optimal regenerative outcomes. Notably, a dECM bioink (hp-bioink) derived from human placental tissue, which met the criteria of printability and bioactivity was introduced [113]. Furthermore, hp-bioink demonstrated the capability to support *in vitro* assembly of HUVECs and promote angiogenesis *in vivo*. Recently researchers introduced a novel printable bioink made from porcine dermis dECM, combined with human dermal fibroblasts to engineer a 3D construct [114]. After a one-week incubation period at 37 °C, the incorporated cells maintained a remarkable survival rate exceeding 90%. Furthermore, microarray analysis unveiled an elevation in the expression of genes implicated in skin repair, underscoring the bioink’s potential for facilitating tissue regeneration [114].

In conclusion, to broaden the applicability of dECM in tissue repair and regeneration, it can be combined with composite biomaterials to fabricate diverse scaffolds tailored to different application scenarios. Scaffolds featuring macro/nanoporosity or nanofibrous structures offer increased binding sites for cell attachment, while the controlled release of dECM promotes the proliferation and differentiation of recruited cells. Furthermore, integrating dECM into hydrogels enables flexible delivery into defect regions or fabrication into various shapes, expanding its potential applications. Beyond mere additives, dECM can also serve as a key ingredient in bioink. Moreover, advancements in decellularization methods and evaluation techniques position dECM scaffolds for promising roles in facilitating vascularized and multilayer functional tissue regeneration [90].

##### Sacrificial materials

**Fig. 11:**
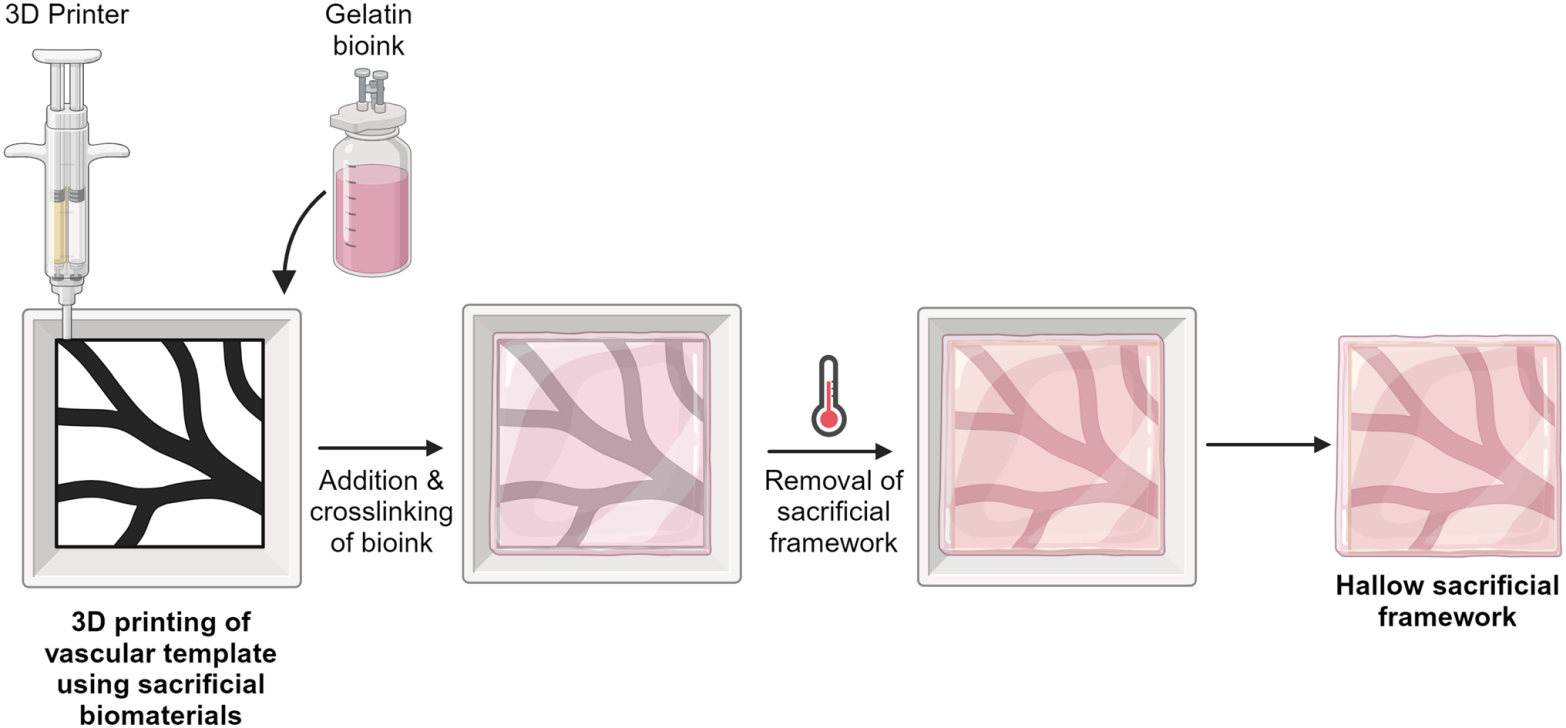
Schematic illustration of 3D printing of vascular network template using sacrificial materials (prepared with BioRender)

Another widely employed method for engineering prevascular networks involves sacrificial materials. Sacrifice-based methods entail removing pre-patterned sacrificial material from the scaffold by leveraging their thermal stabilities, solubilities, or other material properties [115]. A typical fabrication process includes; the first step, a designed sacrificial template is created using methods like 3D printing or casting. Subsequently, the scaffold material, such as gelatin or PDMS, encapsulates the sacrificial template. Upon solidification of the scaffold, the sacrificial template is eliminated by altering environmental parameters, such as temperature or solvent composition. Finally, after thorough rinsing, a scaffold with tissue-engineered vascular networks (TEVs) is fabricated (Fig. 11). The efficacy of these technologies hinges on the selection of sacrificial materials [115], which is further discussed in the subsequent section.

Various sacrificial materials utilized in fabricating prevascular networks can be categorized into four main groups: (1) hydrogel materials; (2) polymeric materials; (3) saccharide-based materials; and (4) customized materials and methods. Despite the diverse array of materials, common manufacturing techniques such as 3D printing and electrospinning are often employed to create intricate sacrificial templates of various sizes. The resulting vascular networks typically exhibit multiscale branched structures.

**Hydrogel sacrificial materials** such as agarose, alginate, and gelatin are commonly utilized in tissue engineering scaffolds owing to their similarity to the ECM [116, 117]. Additionally, these materials can serve as effective sacrificial components in certain applications. Researchers exemplified the utilization of sacrificial agarose fibers embedded within GelMA to construct complex pre-vascular networks [117, 118]. The unique property of agarose, which does not adhere to surrounding photocrosslinkable hydrogels, allows for easy manual removal of the printed sacrificial template. Alginate, another valuable sacrificial material, consists of β-d-mannuronic acid (M), α-l-guluronic acid (G), and alternating M-G residues [118, 119]. While alginate alone lacks mechanical strength for scaffold use, its G-blocks can bind calcium ions, enhancing its mechanical properties when formed into a hydrogel. This hydrogel can then be dissolved using chelators like EDTA and sodium citrate, allowing for the creation of 3D channels in TEVs. Similarly, gelatin serves as a versatile sacrificial material, capable of transitioning from solid to liquid states based on temperature triggers. It solidifies at lower temperatures but liquifies again around 37 °C, offering flexibility in its application for tissue engineering [120, 121] Pluronic F127, a copolymer of poly(ethylene oxide)-poly(propylene oxide)-poly(ethylene oxide) (PEO-PPO-PEO), offers a temperature-triggered phase change mechanism widely used in tissue engineering. Above 10 °C, it acts as a sacrificial template, while below 10 °C, it transforms into a liquid state, allowing for easy removal [122]. Researchers utilized Pluronic F127 in combination with modified Pluronic F127 or GelMA to print 3D microvascular networks. This study innovatively incorporated electrospun PCL fibers as supplementary sacrificial materials to create microscale and nanoscale channels. Together with the macroscale channels formed by removing Pluronic F127, a comprehensive multiscale prevascular network was established [123]. Despite offering a flexible approach for creating fully perfusable and branched prevascular networks, hydrogel sacrificial materials have lingering concerns. At low concentrations, hydrogels, notably alginate, lack self-support due to their weak mechanical properties [124]. To ensure structural integrity, higher concentration alginates are often employed for printing hydrogel-based sacrificial templates. However, the increased concentration of crosslinking agents in these formulations may negatively impact cell viability. Similar challenges arise with Pluronic F127. At high concentrations, it exhibits notable cytotoxicity to cells [125]. Moreover, the low temperatures needed for its removal may harm encapsulated cells [126]. Additionally, its poor mechanical strength poses risks of structural collapse when printing larger constructs [127]. In contrast, gelatin emerges as a promising sacrificial material for prevascular network fabrication due to its phase-changing temperature aligning with cell culture conditions at around 37 °C [115].

**Polymeric sacrificial materials** Polymers represent another prevalent choice for sacrificial materials used for prevascularization in tissue engineering, prized for their excellent processability. Among them, PVA stands out as one of the most utilized [128]. With abundant hydroxyl groups along its molecular chain, PVA boasts excellent water solubility. Its high plasticity allows for easy shaping at its melting temperature (typically 190 °C), facilitating the creation of intricate sacrificial templates. Crucially, PVA exhibits commendable biocompatibility [128]. Mohanty et al. used the 3D PVA sacrificial templates embedded in PDMS yielding prevascular networks upon PVA dissolution in water^129^. Subsequent studies explored PCL as a supportive scaffold for printing PVA, resulting in intricate one-to-four bifurcating structures post-PVA removal [130]. In another study, an L-shaped PLA sacrificial template was printed and embedded into GelMA. Upon PLA removal from the gelation GelMA, a hollow channel remained, forming the basis for a vascular chip [131]. PLA can also be encapsulated in Araldite/Aradur 8605 epoxy, leading to branched vasculature post-vaporization [132]. In addition to their role in constructing macroscale prevascular networks, polymeric sacrificial materials have utility in generating microscale and nanoscale templates through electrospinning. Researchers demonstrated a technique involving the embedding of solvent-spun poly(N-isopropylacrylamide) (PNIPAM) fibers within a crosslinking system containing cell-laden gelatin and microbial transglutaminase to fabricate a 3D microvascular network [133]. PNIPAM fibers were dissolved in the cell culture medium at ambient temperature, eliminating the need for harsh solvents or extreme temperatures during template removal. This approach offered a gentle culture environment for cells within the hydrogel. While 3D microchannels and porous scaffolds have shown promise, concerns persist regarding the biocompatibility of polymers used for sacrificial templates. Removal processes involving high temperatures or solvents can compromise cell viability, posing challenges during scaffold fabrication. Although PVA exhibits biocompatibility, its swelling behavior in water may distort network dimensions. Some studies have addressed this by coating additional layers onto PVA surfaces. However, the mechanical strength of PVA templates remains weaker than other printable polymers, limiting their use in creating small-scale vascular networks < 0.5 mm in diameter [115, 130].

**Saccharide-based sacrificial materials** Saccharides, such as sucrose and maltitol, exhibit favorable characteristics for sacrificial templating in tissue engineering applications. Their inherent biocompatibility and workability make them attractive options for creating intricate structures within scaffold materials [134, 135]. In a study conducted by Lee et al., 3D structures were fabricated using sucrose embedded within PDMS [136]. By dissolving the sucrose at high temperatures, multiscale channels were formed within the PDMS matrix. This method demonstrated the feasibility of utilizing saccharide-based templates to create complex vascular networks within tissue-engineered constructs. Similarly, Wang et al. leveraged sucrose as a sacrificial material for fabricating microfibers via electrospinning [137]. This approach enabled the creation of fine-scale structures suitable for vascularization within tissue scaffolds. He et al. developed a novel 3D sugar printer capable of printing sacrificial structures using maltitol, in conjunction with PDMS as the scaffold material [138]. Upon dissolution of the sacrificial template at elevated temperatures, various types of channels were generated within the PDMS matrix, highlighting the versatility of saccharide-based sacrificial templating [139]. Despite their advantages, saccharide-based sacrificial materials typically require high temperatures for removal. This necessitates careful consideration during cell seeding, as exposure to elevated temperatures may affect cell viability. Additionally, scaffold materials must possess adequate thermal stability to withstand the dissolution process without compromising their structural integrity. An exception to this thermal requirement is Pullulan, a polysaccharide soluble at room temperature. While Pullulan offers the advantage of avoiding high-temperature dissolution, its compatibility with scaffold materials remains an area requiring further investigation. Nonetheless, saccharide-based sacrificial templating holds promise for the fabrication of vascularized tissue constructs in regenerative medicine [115].

**Customized sacrificial materials** The custom fabrication of sacrificial materials presents both innovative opportunities and challenges for TEVs. Miller et al. developed a carbohydrate glass as a biocompatible sacrificial material, enabling the formation of 3D prevascular networks [140]. However, its brittleness may restrict its application for large-scale structures. Tseng et al. synthesized a glucose-sensitive, self-healing hydrogel that dissolves in cell culture medium, offering versatility in sacrificial template fabrication. Yet, its weak mechanical properties pose challenges in controlling dimensional accuracy [141]. Hu et al. introduced an electrolysis-based method to create freestanding 3D microvascular networks using alginate, offering potential for complex vascular structures [142, 143]. However, cell involvement during fabrication remains a challenge due to potential negative effects of metal ions. Furthermore, Guo et al. utilized a pre-mixed sacrificial ink to generate microchannels within PDMS scaffolds, although the high curing temperature limits cell loading [143]. Nazhat et al. developed phosphate-based glass fibers for degradable microchannels within collagen scaffolds, offering tunable degradation rates [144]. However, the demanding fabrication process at ultra-high temperatures hinders practical application efficiency. These custom sacrificial materials demonstrate diverse approaches to TEV fabrication, each with unique advantages and limitations. Future research may focus on refining material properties, improving cell compatibility, and enhancing fabrication efficiency to advance the field of tissue engineering [115].

The challenges and future perspectives in fabricating vascular networks in tissue engineering encompass several key aspects. First, the mechanical properties of engineered scaffolds with vascularized networks remain a concern, as they often do not match those of native tissues. Maintaining shape fidelity during regeneration cycles is crucial, yet weak mechanical properties can lead to scaffold collapse and integration failure with host tissues. Future efforts may focus on developing composite or modified materials to better mimic native tissues and organs, ensuring adequate mechanical support for cell growth and tissue integration. Second, the biocompatibility of sacrificial materials used in TEV fabrication is an ongoing challenge. While sacrifice-based techniques offer promise, non-biocompatible materials, and high-temperature removal methods can impact cell viability and scaffold stability. Utilizing water-soluble sacrificial templates and self-degrading materials may address these concerns, enhancing the flexibility and biocompatibility of fabrication techniques.

Another challenge lies in fabricating TEVs with complex, multiscale, and multilayer structures that mimic native vasculature. Current methods often struggle to replicate the hierarchical branching and layering found in natural blood vessels. Improved precision in printing and fabrication techniques, as well as research into hierarchical scaffold designs, could address this limitation and enhance mass transfer efficiency within engineered constructs. Furthermore, achieving multilayer structures within TEVs remains a significant hurdle. While direct-write techniques offer potential for multilayer fabrication, sacrifice-based methods excel at creating complex 3D vasculatures. Hybrid fabrication techniques combining these approaches may offer a solution, enabling the creation of intricate, multiscale vascular networks. In summary, future directions in TEV fabrication involve enhancing mechanical properties, improving biocompatibility, and advancing fabrication techniques to create complex, customizable vascular networks. This includes developing degradable sacrificial materials, refining hierarchical scaffold designs, and optimizing fabrication processes for clinical applications. By addressing these challenges, next-generation TEVs can offer improved functionality and efficiency for tissue regeneration and vascular research.

### 3D co-culture systems for prevascularization

3D co-culture systems for prevascularization represent a cutting-edge approach in tissue engineering and regenerative medicine aimed at mimicking the complex microenvironment of natural tissues. This strategy involves the co-culturing of multiple cell types within a three-dimensional scaffold to promote the formation of functional blood vessel networks before implantation into the body. In these systems, ECs, which are the building blocks of blood vessels, are typically co-cultured with other relevant cell types, such as pericytes, fibroblasts, or MSCs. The presence of these supportive cell types enhances the stability and functionality of the newly formed blood vessels. One of the key advantages of 3D co-culture systems is their ability to closely mimic the physiological conditions found *in vivo*. By recreating the multicellular interactions and biochemical cues present in native tissue microenvironments, these systems promote the development of robust and mature blood vessel networks within engineered tissues. Moreover, 3D co-culture systems allow for the incorporation of various bioactive molecules, GFs, and ECM components to further enhance vascularization and tissue regeneration processes. This precise control over the microenvironment enables researchers to tailor the properties of the engineered tissues according to specific therapeutic goals and applications.

**Fig. 12:**
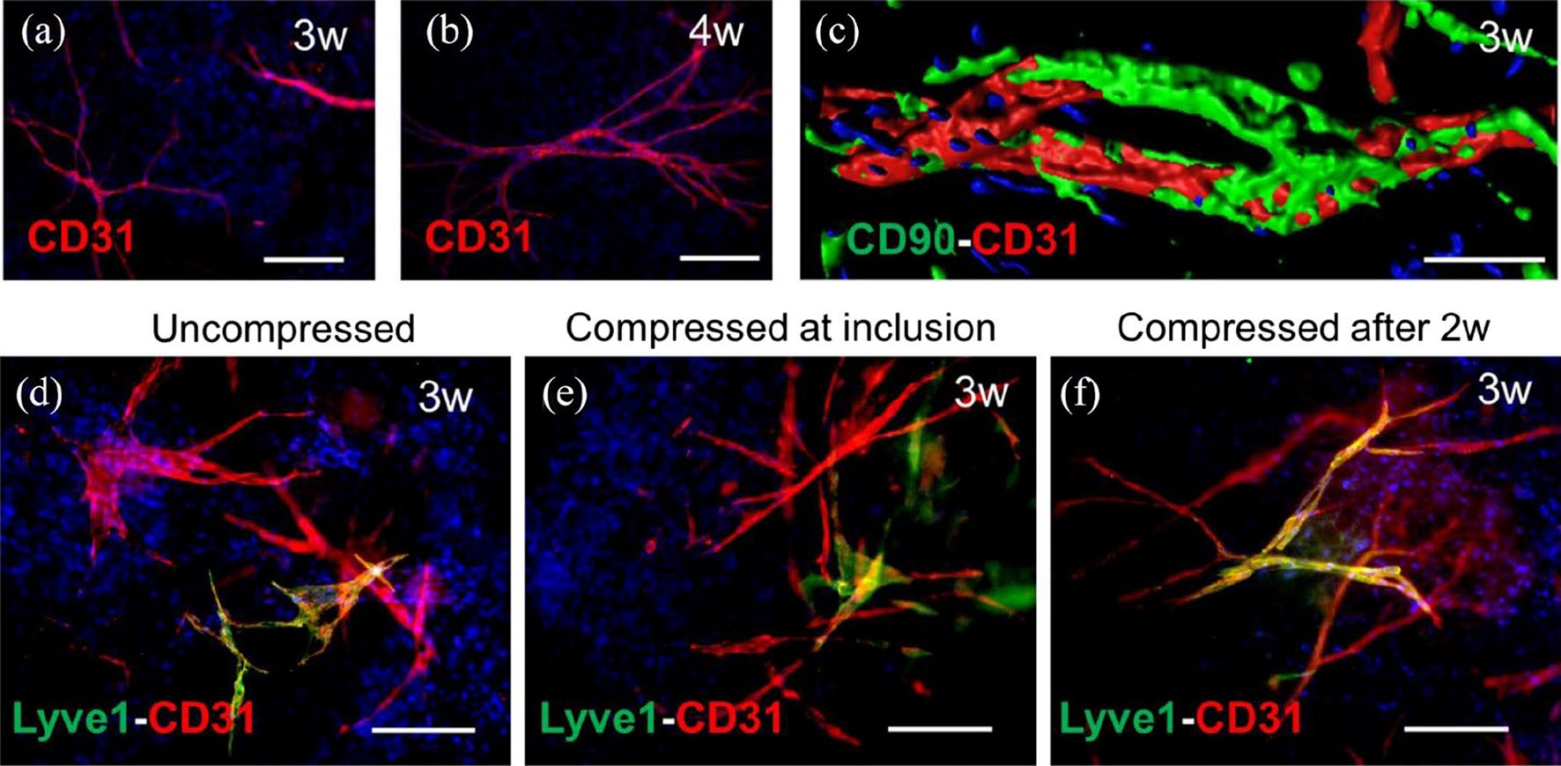
*In vitro* development of prevascularized human DESS by SkinFactory. **a–b** Immunofluorescence whole-mount staining revealed the blood capillaries (CD31) formation in hydrogels after 3 and 4 weeks of HDMECs cultivation via SkinFactory. **c** Detection of mural cells (CD90) around blood capillaries (CD31) after 3 weeks. **d–f** immunofluorescence staining of HDMECs cultured in hydrogels under uncompressed (**d**), compressed on HDMECs seeding (**e**) and compressed after 3 weeks of HDMECs seeding (**f**). Scale bar are 100 µm [modified after [145]

Our laboratory has engineered DESS containing vascular network by applying 3D bioprinting. In particular, using SkinFactory system, fibroblasts and HDMECs were co-seeded within uncompressed collagen type I hydrogels [145]. Subsequently, the cells were transferred to 6 × 6 cm^2^ inserts and cultivated *in vitro* for up to 4 weeks. A mount immunofluorescence staining (Fig. 12) revealed the development of branched vessels expressing CD31 in hydrogels (Fig. 12a–b). Confocal microscopy confirmed the presence of CD90 positive mural cells partially covering the CD31 positive vascular network (Fig. 12c). In order to elucidate compression tolerance of ECs, hydrogels containing HDMECs and vascular network were plastically compressed using SkinFactory. HDMECs developed into vascular (CD31) and lymphatic network (Lyve1) after 3 weeks under both uncompressed conditions (Fig. 12d) as well as compressed conditions (Fig. 12e–f).

**Fig. 13:**
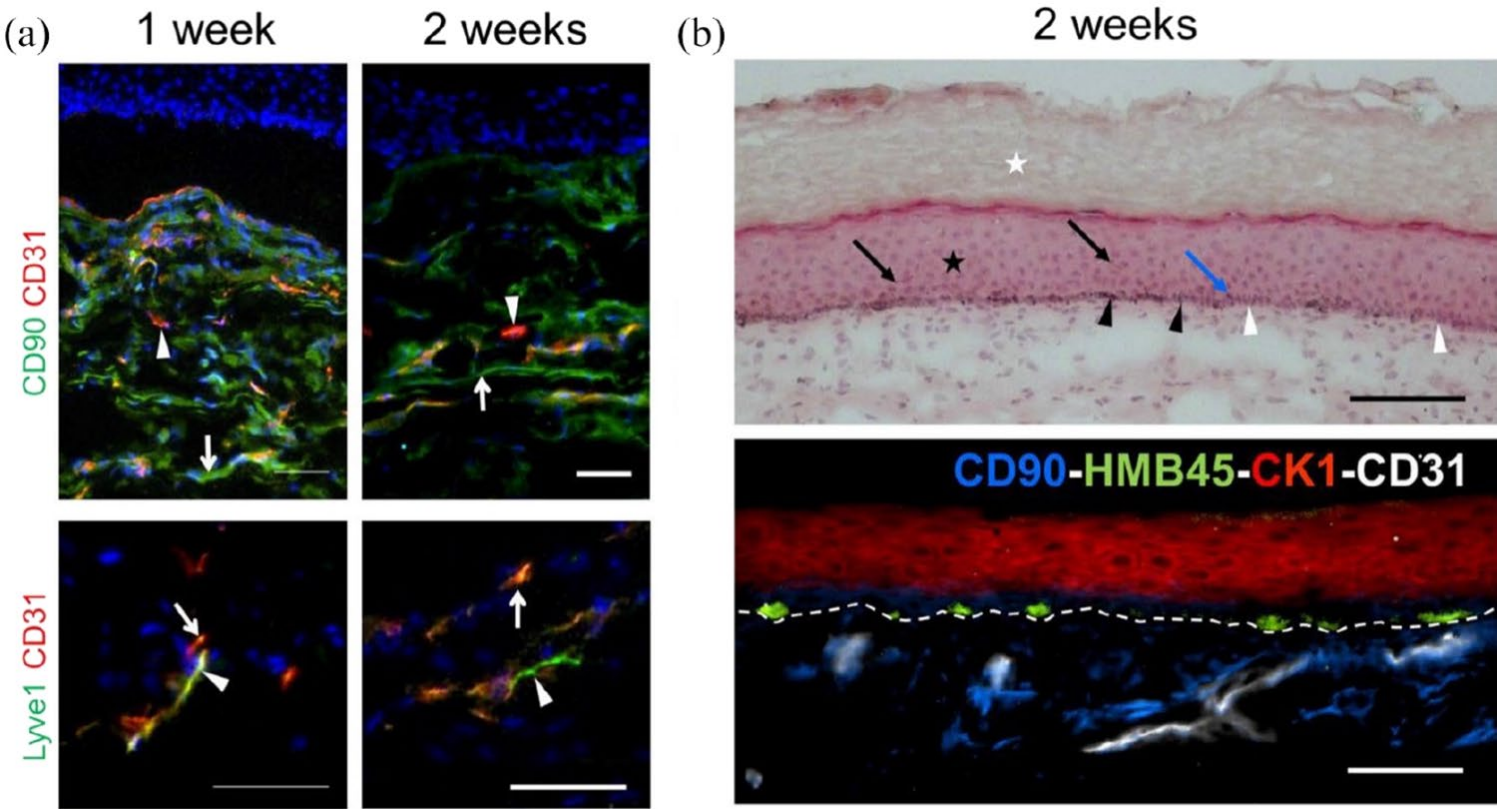
Transplantation of bioprinted prevascularized DESS (pigmented) developed by SkinFactory. **a** Dermal fibroblasts (CD90), blood capillaries (CD31-upper panel) and lymphatic capillaries (Lyve1-lower panel) are visible in neodermis after 1 and 2 weeks of transplantation. **b** Hematoxylin and eosin staining (upper panel) reveals the following structures: the stratum corneum (indicated by a white star), the stratum spinosum (indicated by a black star), pigmented clusters of melanocytes (indicated by black arrowheads), unpigmented clusters of basal keratinocytes (indicated by white arrowheads), melanocyte dendrites (indicated by blue arrows), and melanosome supranuclear caps in keratinocytes (indicated by black arrows). In a four-color immunofluorescence staining, the presence and localization of four distinct cell types are demonstrated: fibroblasts (labeled with CD90 and shown in blue), endothelial cells (labeled with CD31 and shown in white), keratinocytes (labeled with CK1 and shown in red), and melanocytes (labeled with HMB45 and shown in green). The dermo-epidermal border is indicated by the white dotted line. Scale: 200 µm [modified after 145]

Additionally, we successfully combined pigmentation with prevascularized DESS “PV-DESS” using SkinFactory. For development of PV-DESS, keratinocytes with melanocytes were added in epidermal compartment along with HDMECs and fibroblasts in dermal compressed compartment. Newly developed PV-DESS was transplanted in animal model for evaluation of its functionality. After 1 and 2 weeks of transplantation, immunofluorescence staining confirmed the presence of human capillary network using the human CD31 and human CD90 (Fig. 13a). Interestingly, immunofluorescence double staining also demonstrated a network of lymphatic capillaries (Fig. 13a–lower panel) using human CD31 and human LYVE1. Quality of PV-DESS after transplantation was confirmed by hematoxylin/eosin staining (Fig. 13b), revealing the presence of cornified layer, stratification, and basal keratinocytes along with melanocytes. Further, a four-color immunofluorescence staining (Fig. 13b) confirmed the localization of all used human cell types as fibroblasts (CD90, blue), melanocytes (HMB45, green), keratinocytes (CK1, red), and ECs (CD31, white) [145].

In conclusion, 3D bioprinting allows rapid generation of tissues/organs on-demand, which is highly convenient for researchers and clinicians. It allows for the creation of custom tissues as needed, reducing the need for extensive storage and transportation of donor organs. However, one of the major challenges in bioprinting of tissue-engineered tissues/organs is to increase miniaturization of *in vitro* vasculature, which needs to be close to capillary size. The production of such small-size microchannels still presents certain technical difficulties, however the main challenge remains endothelializing those channels due to high numbers of detached ECs in comparison to the channel diameter. Therefore, more studies are required to improve protocols for EC culture in microfluidics, particularly for the conditions required to promote EC viability. Currently HUVECs are the most common type of cell employed for *in vitro* microvasculature due to their high availability and established techniques, especially since they create sturdy channels. Therefore, more research is needed to apply also ECs from other tissue sources. However, these cells can be challenging to culture in microfluidics, since only a few studies are available in this field [145]

### miRNA modulation for therapeutic angiogenesis

miRNA modulation represents an alternative method to enhance the vasculogenic potential of cells. Small, non-coding RNA segments called miRNAs play a significant role in the regulation of gene expression by accelerating messenger RNA disintegration and preventing it from being translated. Further, miRNAs have a high half-life and can be overexpressed or silenced, which allows for a continuous modification of cell activity.

The miR-17/92 family comprises six microRNAs: miR-17, miR-18a, miR-19a, miR-20a, miR-19b-1, and miR-92a-1 [146]. Studies have demonstrated that animals lacking this microRNA family die postnatally from a ventricular septal defect. This family has a high level of expression in ECs, and by obstructing EC motility, it prevents angiogenesis. The miR-17/92 family of microRNAs controls the expression of proto-oncogenes and inhibits the expression of thrombospondin and connective tissue GF, and anti-angiogenic molecules. Numerous studies have been conducted on the individual members of this family of microRNAs. For instance, miR-92a has been shown to have a positive effect on the development of blood vessels in ischemic injury and myocardial infarction [147].

Considering this, miRNA manipulation has recently been proposed as a particularly viable method for improving the therapeutic effectiveness of skin analogs. miRNAs play a crucial role in the maintenance of angiogenesis and EC activity, which raises the possibility of using exogenous miRNAs to increase vascularization in skin tissue engineering. This can be achieved by preconditioning wounds to miRNAs to enhance the physical state of the wounded area and, as a result, make it easier for skin substitutes to engraft. A promising option for improving wound angiogenesis is MiR-126, which has significant expression in cutaneous ECs and improves the effectivity of FGF and VEGF on blood vessel formation. In addition, particular miRNAs may be transfected into ECs of skin substitutes to enhance their angiogenic capabilities. The recent work by Devalliere et al*.* provides an intriguing illustration of such a strategy [148]. They subcutaneously implanted collagen-fibronectin gels containing HUVEC that had been miR-132 transfected. When compared to control transfected samples, the implants had more mural cell-covered vessels and a greater micro-vessel density [148].

### Scaffold-free cell sheet engineering for angiogenesis stimulation

Cell sheet technology is an innovative approach in regenerative medicine and tissue engineering, aimed at creating a network of blood vessels within engineered tissues before implantation. The scaffold-free manufacturing of artificial skin made of multi-layered cell sheets presents an interesting alternative to avoid problems with cell seeding effectiveness or biocompatibility that are frequently associated with traditional scaffold-based approaches. Recently, the research focused on the development of a monolayer of the cell on a surface coated with temperature-sensitive poly-(N-isopropylacrylamide) [149]. At temperatures below 32 °C, this surface rapidly hydrates and expands, releasing a confluent cell sheet that has functional cell-cell connections, releasing a deposited ECM from the cultured cells [149].

Under the right culture conditions, cell sheets’ ability to vascularize can be increased. For instance, Yu et al*.* [150] activated fibroblast cell sheets in vitro using bioglass. This bioactive silicate component stimulated the secretion of several pro-angiogenic GFs by the fibroblasts. Thus, the implantation of the activated cell sheets in full-thickness skin defects of mice significantly boosted collagen synthesis, angiogenesis, and myofibroblasts development from fibroblast differentiation on the damage site as compared to the non-active control sheets. On the other hand, MSCs may be employed to create cell sheets since they are known to have a significant intrinsic pro-angiogenic potential [151]. Furthermore, ECs have been demonstrated to naturally establish single layers and sandwiched cell sheets of microvascular networks [152], indicating that prevascularized skin substitutes may be engineered. By combining cell sheets containing HDMECs, fibroblasts, and keratinocytes, Cerqueira et al*.* [153] recently demonstrated enhanced vascularization, re-epithelialization, and ECM remodeling during skin healing.

Although there are commercially accessible skin and cartilage tissue products, most are only available in thin tissues. Enhancing therapeutic effectiveness for the treatment of serious diseases may be possible through the engineering of larger and more complex tissues. Cell sheet technology allows for the creation of tissue constructs without the need for external scaffolds or foreign materials. This reduces the risk of immune responses or foreign body reactions often associated with synthetic scaffolds. It can lead to the rapid formation of three-dimensional tissues with substantial volumes. This is especially advantageous for producing large tissue constructs and organs. Unlike some tissue dissociation methods, cell sheet technology preserves cell-cell and cell-ECM interactions without the need for enzymatic digestion. This minimizes potential damage to the cells and their microenvironments. Cell sheet constructs can achieve higher cell densities, which can be important for tissue function and integration upon transplantation. This density promotes a more natural and functional tissue structure. Cell sheets often have a uniform distribution of cells, ensuring that all areas of the tissue construct receive a consistent supply of cells and nutrients. This uniformity can improve tissue quality and functionality. This technology allows for the preservation of cell-matrix interactions, promoting the formation of a native-like ECM and enhancing cell signaling and communication [154].

Besides advantages, this technique offers several disadvantages, which are essential to consider for its successful implementation. For example, cell sheet technology may not always promote the upregulation of angiogenesis factors necessary for the formation of blood vessels within the tissue construct. This limitation can affect the vascularization and nutrient supply to the engineered tissue. The process of producing cell sheets can be labor-intensive and time-consuming. It involves multiple steps, including cell culture, sheet manipulation, and stacking, which can be resource-intensive and may limit scalability. Cell sheets are generally more suitable for creating thin tissues. In contrast, producing thicker tissues or whole organs using cell sheet technology can be challenging, as diffusion limitations can hinder nutrient and oxygen supply to inner cells. Cell sheet technology is sensitive to temperature changes. Fluctuations in temperature during cell sheet harvesting, manipulation, or transplantation can impact cell viability and functionality, making temperature control crucial [155].

 Therefore, researchers are actively working to address these limitations through various approaches, such as improving vascularization strategies, automating production processes, and enhancing temperature control methods, to make cell sheet technology more versatile and applicable in a wider range of tissue engineering scenarios. In conclusion, cell sheet technology for prevascularization offers promising benefits for tissue engineering. However, it also comes with challenges related to complexity, standardization, and suitability for different tissue types. Future research and technological advancements may help address some of these disadvantages and further enhance the effectiveness of this approach.

## Summary

The rapid development of a microvascular network and establishment of functional blood perfusion is crucial for the successful integration of skin grafts and dermal templates. Thus, to improve skin regeneration, understanding the fundamental principles underlying the prevascularization of skin substitutes is essential, as to utilize these principles into the clinical settings. Indeed, various methods have been investigated over the past few decades to enhance skin vascularization, which is crucial for the proper healing of major wounds. There are currently three ways to activate biological scaffolds; altering the structural and physicochemical characteristics of dermal scaffolds; using GF-releasing systems or gene vectors to activate biological scaffolds; and creating prevascularized skin substitutes by integrating capillary-forming cells into scaffolds.

In addition to these traditional methods, novel approaches including 3D bioprinting, miRNA editing, microfluidics, and grafting of microvascular segments derived from adipose tissue may be implemented. Further, pro-angiogenic GFs have been utilized to enhance vascular formation, however controlling the spatiotemporal and sequential release of several GF is complicated. Therefore, new approaches have been established using GF-releasing stem cells within the scaffolds. However, still the low and not timely controllable secretion of GF from those cells may limit the angiogenesis response, since after an injury, levels of pro-angiogenic factors increase, reaching a peak slightly before maximum capillary content occurs, and then subside to nearly undetectable levels. In addition, multiple GF need to be present to promote wound angiogenesis. Further, the selective coverage of capillaries by pericytes plays an important role in capillary maturation, by increasing vascular stability over time.

It is also necessary to enhance the mechanical and physical properties of biological scaffolds. Multiple combination technologies may be combined to create synthetic vascular tissue substitute for clinical applications. Taken together, the developments specially related to skin substitute vascularization have recently shown the rapid advancements in tissue engineering and regenerative medicine. The development of novel vascularization techniques combined with ongoing research into the fundamental processes behind blood vessel formation opens the door to novel therapies.

## Conclusion

Majority of above-mentioned vascularization methods are insufficient to promote high engraftment rates of tissue-engineered skin constructs. Therefore, novel approaches to ensure optimal therapeutic outcomes following skin replacement are still required. Current research strategies focus on combining prevascularization and angiogenesis-promoting techniques, as a therapeutic modality accelerating the healing of wounds. Such a combinational strategy is anticipated to speed up the process of engrafting of the prevascularized skin grafts or dermal templates into the host tissue. Further, the lymphatic system, which has been successfully integrated together with blood microvasculature helps to remove interstitial fluid and reduce edema. Thus, a healthy lymphatic system is essential for the engraftment of skin replacements and currently therapeutic lymphangiogenesis is a new area of research in skin repair. Further the sensory system, which is known to be essential for the perception of heat, pain, and touch, should also be integrated within the vascularized structures in order to accurately mimic the complex composition and architectural arrangement of skin tissues.

Successful in vitro vascularization requires a vascular pedicle connecting the engineered microvascular network to the perfusion reactor and/or host internal circulatory system. Therefore, only a functional microvascular network promotes rapid graft intake and longterm survival. However, in order to develop complex engineered vascular tissues for clinical use, it is imperative to establish multiple combination strategies. Therefore, a combined approach using 3D-bioprinting, perfusion reactors, and tissue-engineering is crucial to successfully produce prevascularized skin tissues for clinical use.
